# Tumor necroptosis is correlated with a favorable immune cell signature and programmed death-ligand 1 expression in cholangiocarcinoma

**DOI:** 10.1038/s41598-021-89977-9

**Published:** 2021-06-03

**Authors:** Thanpisit Lomphithak, Perawatt Akara-amornthum, Keigo Murakami, Masatoshi Hashimoto, Hajime Usubuchi, Erina Iwabuchi, Michiaki Unno, Zhenyu Cai, Hironobu Sasano, Siriporn Jitkaew

**Affiliations:** 1 Graduate Program in Clinical Biochemistry and Molecular Medicine, Department of Clinical Chemistry, Faculty of Allied Health Sciences, Chulalongkorn University, Bangkok, 10330 Thailand; 2 Department of Pathology, Tohoku University School of Medicine, Sendai, Miyagi 980-8575 Japan; 3 Department of Surgery, Tohoku University School of Medicine, Sendai, Miyagi 98-8075 Japan; 4 Tongji University Cancer Center, Shanghai Tenth People’s Hospital, School of Medicine, Tongji University, Shanghai, 200072 China; 5 Age-Related Inflammation and Degeneration Research Unit, Department of Clinical Chemistry, Faculty of Allied Health Sciences, Chulalongkorn University, Bangkok, 10330 Thailand

**Keywords:** Tumour immunology, Cell death and immune response, Immune cell death, Immunotherapy, Inflammation, Tumour immunology

## Abstract

Necroptosis, a regulated form of necrosis, has emerged as a novel therapeutic strategy that could enhance cancer immunotherapy. However, its role in tumorigenesis is still debated because recent studies have reported both anti- and pro-tumoral effects. Here, we aimed to systematically evaluate the associations between tumor necroptosis (mixed lineage kinase domain-like protein, MLKL; phosphorylated MLKL, pMLKL; and receptor-interacting protein kinase 1–receptor-interacting protein kinase 3, RIPK1–RIPK3 interaction) and tumor-infiltrating immune cells (CD8+ and FOXp3+ T cells and CD163+ M2 macrophages) and tumor PD-L1 by immunohistochemistry in 88 cholangiocarcinoma (CCA) patients who had undergone surgical resection. Their associations with clinicopathological characteristics, survival data, and prognosis were evaluated. MLKL was found to be an unfavorable prognostic factor (*p-*value = 0.023, HR = 2.070) and was inversely correlated with a clinically favorable immune cell signature (high CD8+/high FOXp3+/low CD163+). Both pMLKL and RIPK1–RIPK3 interaction were detected in CCA primary tissues. In contrast to MLKL, pMLKL status was significantly positively correlated with a favorable immune signature (high CD8+/high FOXp3+/low CD163+) and PD-L1 expression. Patients with high pMLKL-positive staining were significantly associated with an increased abundance of CD8+ T cell intratumoral infiltration (*p*-value = 0.006). Patients with high pMLKL and PD-L1 expressions had a longer overall survival (OS). The results from in vitro experiments showed that necroptosis activation in an RMCCA-1 human CCA cell line selectively promoted proinflammatory cytokine and chemokine expression. Jurkat T cells stimulated with necroptotic RMCCA-1-derived conditioned medium promoted PD-L1 expression in RMCCA-1. Our findings demonstrated the differential associations of necroptosis activation (pMLKL) and MLKL with a clinically favorable immune signature and survival rates and highlighted a novel therapeutic possibility for combining a necroptosis-based therapeutic approach with immune checkpoint inhibitors for more efficient treatment of CCA patients.

## Introduction

Cholangiocarcinoma (CCA) is a highly heterogeneous malignancy that could arise at any level of the biliary tree. CCA is classified into intra- (iCCA) and extra- (eCCA) hepatic cholangiocarcinoma in which eCCA can be further subdivided into perihilar (pCCA) and distal (dCCA)^1^. Despite recent advances in the development of early detection and identification of novel therapeutic targets in several human malignancies, most CCA patients are typically diagnosed at advanced clinical stages, and treatment options are enormously limited because overall survival rates have by no means been improved^2^.

The interaction between tumor and inflammatory/immune cells and chronic inflammation in the tumor microenvironment (TME) has been considered to play critical roles in tumorigenesis, progression, recurrence, and cancer patients’ therapeutic responses^3^. Infiltration of inflammatory and immune cells and their impact on patient outcomes have been continuously reported in CCA. High infiltration of tumor-infiltrating CD8+, CD4+, and FOXp3+ regulatory (Tregs) T cells and low CD163+ M2 macrophages (TAMs) were found to be significantly correlated with a longer overall survival in eCCA patients^4^. In addition, an immune high-risk signature characterized by high tumor-associated neutrophils (TANs), high FOXp3+, and low CD8+ T cells was recently reported to be significantly associated with poor disease-free and overall survival rates (DFS and OS) and resistance to gemcitabine treatment after recurrence in eCCA patients^5^. Therefore, immunotherapy was proposed as a potential strategy for the treatment of CCA patients^2,6^. In addition, a particular subgroup of CCA patients represented as an inflamed subtype generally characterized by a massive T cell infiltration, inflammatory and immune checkpoint activation was proposed to potentially benefit from checkpoint blockade immunotherapy^7^. Therefore, systemic analysis toward a better understanding of the interplay between inflammatory/immune cells and their responses, particularly immunogenic dying cells in TME, should lead to the novel development of more efficient cancer immunotherapy.

Necroptosis is a regulated form of cell death in a caspase-independent manner. In contrast to apoptosis, necroptosis is known to elicit marked inflammatory responses and adaptive immunity. Upon necroptosis activation, receptor-interacting protein kinase 1 (RIPK1) interacts with receptor-interacting protein kinase 3 (RIPK3) through their RIP homotypic interaction motif (RHIM) domains, resulting in the formation of a necrosome complex^8^. In this complex, the mixed lineage kinase domain-like protein (MLKL) is phosphorylated by RIPK3, subsequently leading to its oligomerization and translocation to the plasma membrane, resulting in rapid membrane permeabilization^9–11^ and the release of intracellular contents, including damage-associated molecular patterns (DAMPs)^12^.

Accumulating studies have demonstrated that necroptosis could play important roles in several human diseases, including cancers. Necroptosis in the spectrum of immunogenic cell death (ICD) has also been proposed as a promising novel cancer therapy^13–16^. On the one hand, necroptosis evokes strong adaptive immune responses and, therefore, may trigger and enhance anti-tumor immunity and cancer immunotherapy; on the other hand, the associated inflammatory responses could also promote cancer development and progression. Necroptosis of endothelial cells in the tumor microenvironment has been reported to promote tumor cell extravasation and metastasis^17,18^, and necroptosis of tumor cells can also promote cancer metastasis^19^. RIPK3 depletion could generate an immunosuppressive tumor microenvironment and tumorigenesis in pancreatic adenocarcinoma (PDA) experimental mouse model^20^. A recent study demonstrated that necroptosis in hepatic microenvironments can direct lineage commitment to switch from hepatocellular carcinoma (HCC) to CCA^21^. In addition, radiation-induced necroptosis was also reported to contribute to tumor cell repopulation and recurrence in colorectal cancer^22^. However, it is also true that necroptosis was found to function as a tumor suppressor, although its mechanisms have remained virtually unknown^23,24^. Most necroptosis in cancer, however, has been reported in vitro and/or animal models, but it is entirely true that these studies are enormously limited by the lack of comparative evaluation in clinical settings.

Therefore, in this study, we systematically analyzed the associations of key necroptotic proteins, necroptosis activation, PD-L1, tumor-infiltrating immune cells and their impact on clinical outcomes and anti-tumor immunity in CCA patients. In addition, in vitro experiments were performed to provide the possible underlying mechanisms of tumor necroptosis-promoted immune cell infiltration and PD-L1 expression. Our results provide valuable information toward a better understanding of necroptosis, MLKL in CCA, and for a potential cancer immunotherapy as well as novel prognostic markers.

## Results

### Higher expression of MLKL is associated with poor survival rates in CCA patients

**Figure 1:**
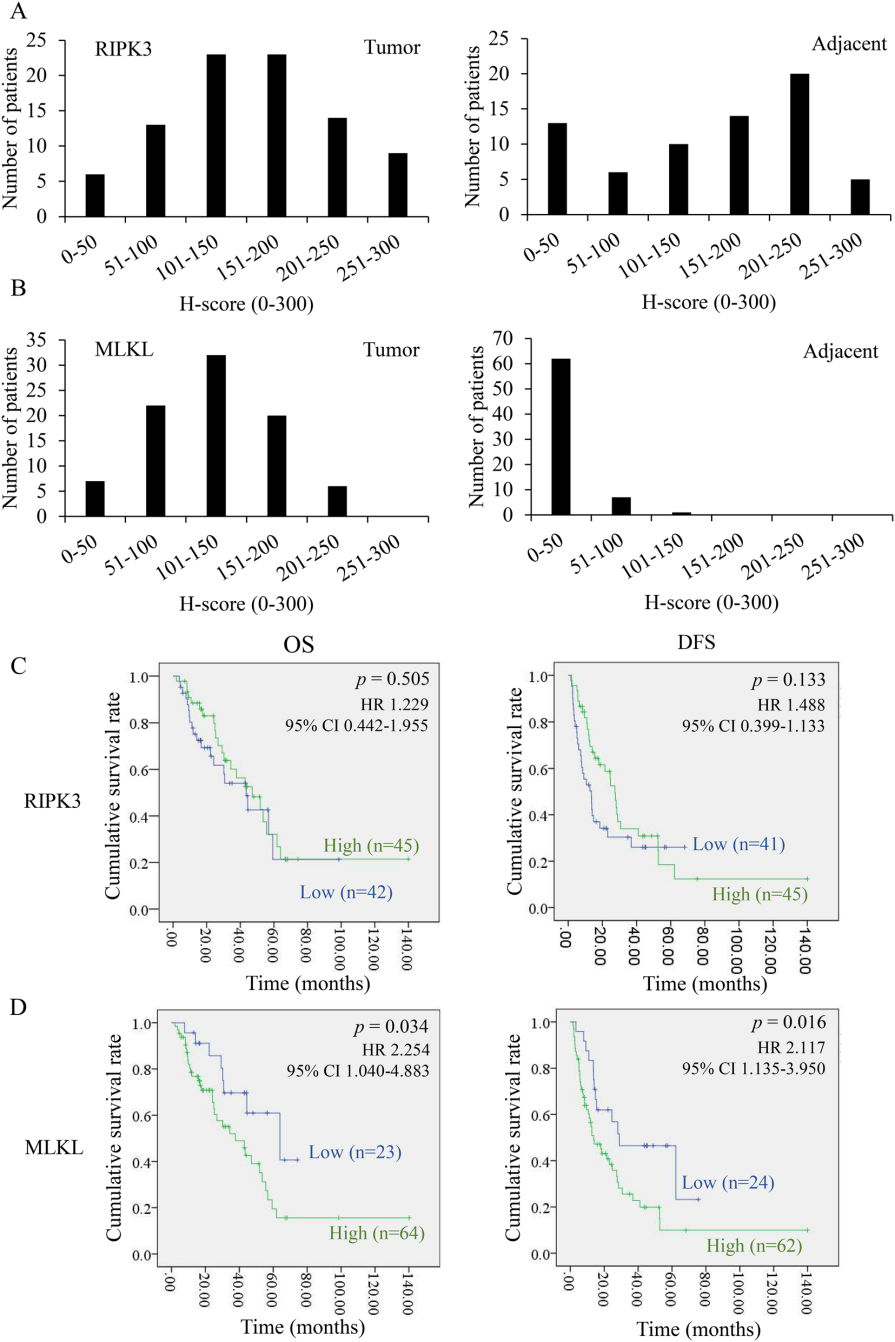
Key necroptotic protein expression and the associations with survival rates in CCA patients. Distributions of RIPK3 (**A**) and MLKL (**B**) expression levels according to H-score of tumor and adjacent area. Kaplan–Meier overall (OS) and disease-free survival (DFS) curves divided by the median of H-score of RIPK3 (**C**) and MLKL (**D**) expression (high vs low).

We attempted to study the in vivo relevance of RIPK3 and MLKL protein expression in clinical cases of CCA^25^. RIPK3 immunoreactivity was differentially present and mainly localized in the cytoplasm of CCA tissues, normal cholangiocyte adjacent to tumor tissues, and cholangiocytes from normal liver tissues (Fig. 1A, Supplementary Fig. S1A). MLKL was also mainly immunolocalized in the cytoplasm of CCA tissues, normal cholangiocyte adjacent to tumor tissues, and cholangiocytes in normal liver tissues (Fig. 1B, Supplementary Fig. S1B). The status of MLKL immunoreactivity was significantly higher in both hilar (n = 68) (*p-*value = 1.1394E−24) and intrahepatic CCA tissues (n = 21) (*p-*value = 7.8992E−10) than adjacent tumor tissues (Supplementary Fig. S1C). Of particular importance, RIPK3 was moderately expressed in both CCA and adjacent tumor tissues with a median H-score of 158.5 and 154.9, respectively (Supplementary Fig. S1C). However, that of MLKL in both hilar and intrahepatic CCA was much lower than that of RIPK3 in adjacent tumor tissues, in which 88.7% of the patients had a median H-score of 22.03, which was subsequently interpreted as negative (Supplementary Fig. S1C). The Kaplan–Meier survival curves revealed no significant association between RIPK3 and OS and DFS (Fig. 1C). In marked contrast, MLKL was significantly associated with both shorter OS and DFS in CCA patients (Fig. 1D). These results indicated that both RIPK3 and MLKL were differentially detected in human CCA primary tissues, but only MLKL was significantly upregulated in tumor tissues in which high MLKL expression was associated with poor survival rates in CCA patients.

### MLKL is an unfavorable prognostic factor for patients with CCA

Univariate and multivariate analyses of DFS and OS were subsequently performed to identify independent prognostic factors in patients with combined hilar and intrahepatic CCA using a Cox proportional-hazard model. On univariate analysis, CCA subtypes, vascular invasion, and MLKL expression were associated with a shorter DFS (Table 1), and vascular invasion and MLKL expression were associated with a shorter OS (Supplementary Table S2). In the multivariate analysis, only MLKL was shown to be an independent prognostic factor for DFS (Table 1). These results suggested that MLKL was an unfavorable prognostic factor for patients with CCA (*p-*value = 0.023, HR = 2.070).

**Table 1:** Univariate and multivariate Cox proportional hazard analysis for disease-free survival of CCA patients.

Factor	Univariate				Multivariate			
	95% CI		HR	*p*-value	95% CI		HR	*p*-value
Gender	0.745	2.177	1.274	0.376				
Age (MED = 67 years)	0.797	2.602	1.440	0.227				
Type	1.322	4.173	2.349	0.004	0.255	15.347	1.979	0.514
HistoGradeEU	0.188	1.040	0.442	0.061				
Tumor size (MED = 35 mm)	0.842	2.703	1.508	0.167				
RIPK3 (MED_H-Score_ = 160)	0.883	2.507	1.488	0.136				
MLKL (MED_H-Score_ = 120)	1.135	3.950	2.117	0.018	1.106	3.872	2.070	0.023
Vascular invasion	0.273	0.848	0.481	0.011	0.586	33.958	4.461	0.149
Neural/perineural invasion	0.354	1.102	0.625	0.104				
Lymph node invasion	0.364	1.110	0.636	0.111				
TNM stage	0.547	1.625	0.942	0.831				

### Necroptosis is activated in human CCA patients

**Figure 2:**
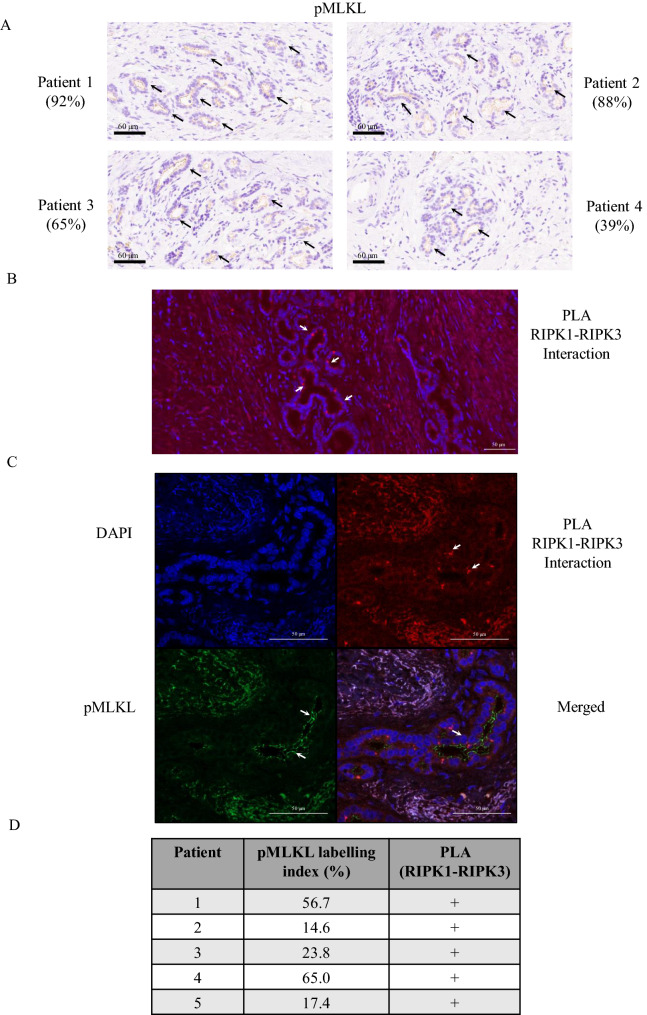
Necroptosis detection in human CCA primary tissues. (**A**) The representative membranous pMLKL immunostaining in primary CCA tissues. Black arrowheads indicate bile ducts and pMLKL positive staining. Four representative images obtained from four CCA patients with different pMLKL labeling index including 1. 92%, 2. 88%, 3. 65%, and 4. 39%, respectively. (**B**) The representative of positive signal (red dots) for RIPK1-RIPK3 interaction in primary CCA tissues using PLA assay. (**C**) The representative of double staining for pMLKL immunofluorescence staining and RIPK1-RIPK3 using PLA and in primary CCA tissues. White arrowheads indicate bile ducts and positive staining (**D**) The pMLKL and PLA (RIPK1-RIPK3) status of 5 selected CCA patients.

Recent studies have reported that the MLKL protein might function independently of necroptosis activation in cancer^26^. To determine whether necroptosis is activated and contributes to the survival rates of CCA patients, phosphorylated MLKL (pMLKL) and RIPK1–RIPK3 interaction were developed to detect necroptosis activation in human primary tissues. First, we optimized and validated the specificity of pMLKL antibody that recognizes MLKL phosphorylation at serine 358 using immunohistochemical (IHC) and immunofluorescence (IF) staining. HT-29 colon cancer cells were treated with TNF-α/Smac mimetic/zVAD-fmk (TSZ) to induce necroptosis^10^, and cells were solidified in iPGell and embedded in paraffin in a routine manner. Phosphorylated MLKL was predominantly stained around cell membranes in the TSZ treatment, but there was no staining in control cells (Supplementary Fig. S2A,B). We used this validated pMLKL antibody to investigate the expression of pMLKL in human CCA primary tissues in which cholangiocytes and CCA cells were evaluated by pathologists based on their morphology using H&E staining. Interestingly, pMLKL was detected in human CCA cells and predominantly localized at the plasma membranes (Fig. 2A). Of note, we observed that CCA cells with pMLKL-positive staining indicating necroptotic cells were not presented in the tumor necrosis area, although we cannot exclude the possibility that a high degree of tumor necrosis was not stained by the pMLKL antibody. Moreover, RIPK1–RIPK3 interaction was analyzed by an in situ proximity ligation assay (PLA). PLA detection of RIPK1–RIPK3 interaction was optimized in iPGell-solidified/paraffin-embedded HT-29 cells as mentioned above. The positive signal represented by red dots reflecting RIPK1–RIPK3 interaction was observed in the cytoplasm of TSZ-treated HT-29 cells (Supplementary Fig. S2C). Similar to pMLKL, RIPK1–RIPK3 interaction was detected in human CCA primary tissues in which red dots are mainly localized in CCA cells (Fig. 2B). Furthermore, we performed IF double staining by combined pMLKL IF staining with RIPK1–RIPK3 interaction via a PLA assay. Interestingly, both pMLKL and RIPK1–RIPK3 interaction were detected in human CCA cells (Fig. 2C). Five of the CCA primary tissues that were positive for pMLKL staining were also positive for RIPK1–RIPK3 interaction according to the PLA assay (Fig. 2D). Our in vivo necroptosis detection methods demonstrated that necroptosis activation was detected in human CCA primary tissues.

### Higher pMLKL is associated with better OS and DFS in CCA patients

**Figure 3:**
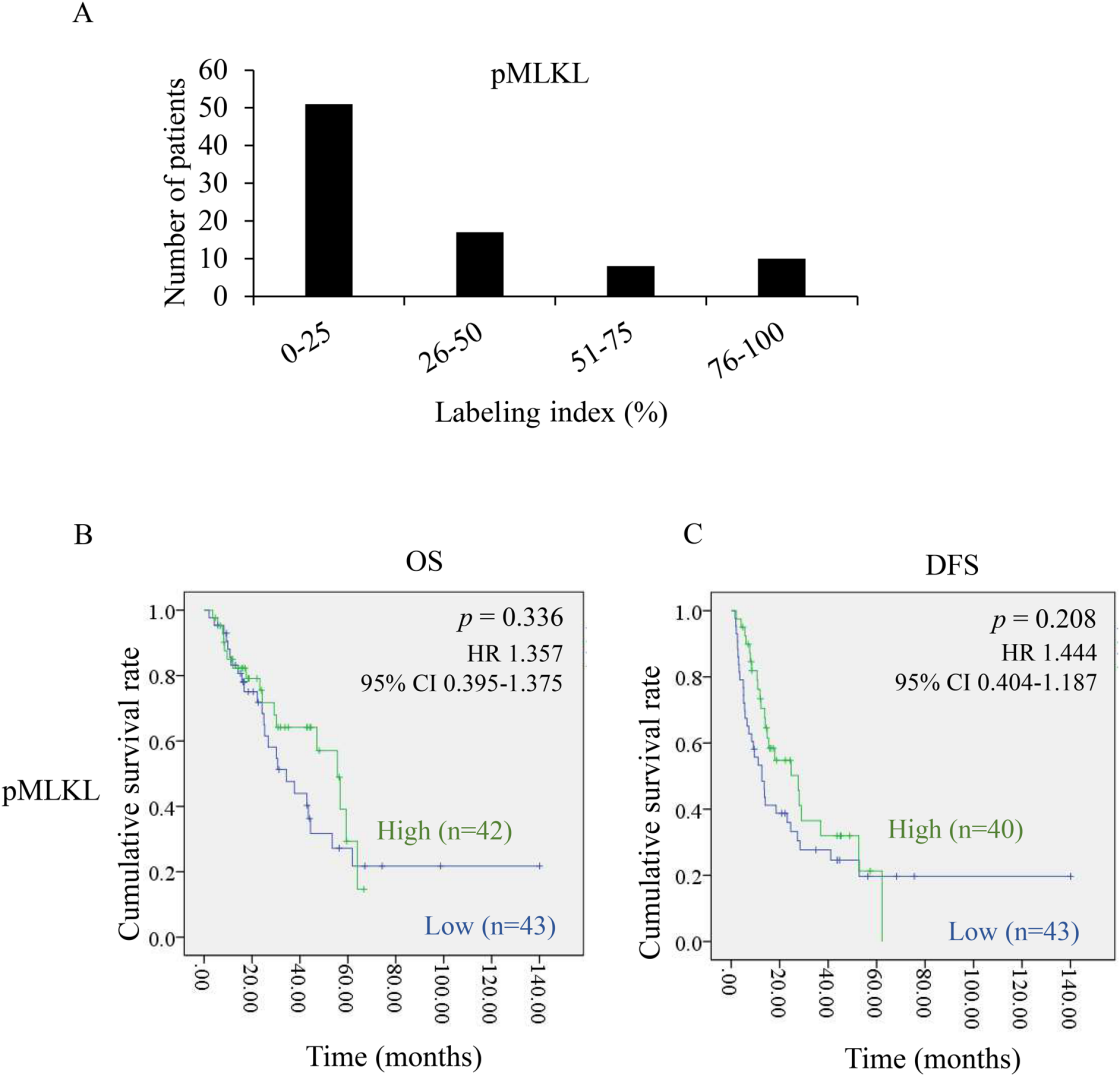
Necroptosis activation and its association with survival rates in CCA patients. (**A**) Distribution of pMLKL expression according to % labeling index calculation. (**B**) Kaplan–Meier overall (OS) and disease-free (DFS) survival curves divided by the median of % labeling index of pMLKL expression.

We next performed IHC staining of pMLKL in a total of 88 primary CCA specimens and evaluated the association of pMLKL with survival time of CCA patients. The percentage of pMLKL labeling index was differentially detected in human CCA primary tissues (Fig. 3A) in which 50.6% of the patients with a median labeling index of 21.30 was interpreted as high pMLKL expression. Kaplan–Meier survival curves showed a trend of high pMLKL associated with better OS and DFS. The median of OS in patients with high pMLKL was 55 months compared with that of 34 months for patients with low pMLKL (Fig. 3B). Similarly, the median of DFS in patients with high pMLKL and low pMLKL was 27 months and 16 months, respectively (Fig. 3C). Altogether, these results indicated that necroptosis was detected in CCA primary tissues and had a trend of a longer OS and DFS in CCA patients.

### The distribution of tumor-infiltrating immune cells and their impact on patient survival rates

**Figure 4:**
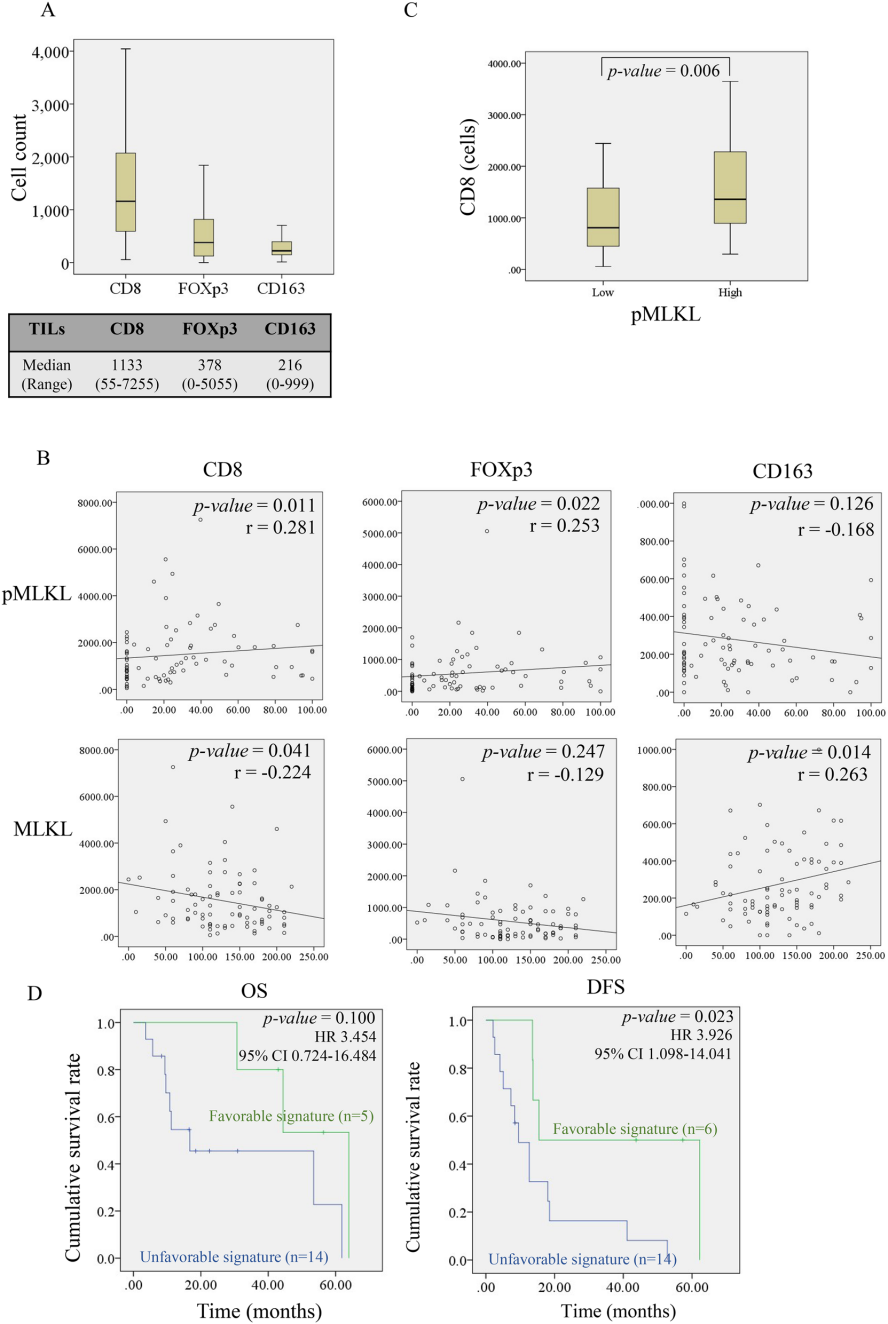
Differential associations of necroptosis activation and MLKL with inflammatory/immune cells infiltration. (**A**) Box plots showing the distributions of tumor-infiltrating CD8+ T cells, FOXp3+ T cells, and CD163+ M2 macrophages. (**B**) The correlation analysis of necroptosis activation (pMLKL) and MLKL with CD8+ T cells, FOXp3+ T cells, and CD163+ M2 macrophages. (**C**) Boxplot representing the relationship between the infiltration of CD8+ T cells in tumor central area and pMLKL positive staining (Mann–Whitney *U*-test). (**D**) Kaplan–Meier survival analysis of CCA patients divided by a favorable MLKL/immune signature (high CD8+ /high FOXp3+ /low CD163+ /low MLKL) and an unfavorable MLKL/immune signature (low CD8+ /low FOXp3+ /high CD163+ /high MLKL) for overall (OS) and disease-free (DFS) survival rates.

The abundance of inflammatory/immune cell infiltration, an indicator of chronic inflammation, was found in the CCA microenvironment (Supplementary Fig. S3A,B). Tumor-infiltrating inflammatory/immune cells can be classified into diverse subsets with antagonistic functions^27^. We used IHC staining to evaluate the infiltration of tumor-infiltrating inflammatory/immune cells in the CCA microenvironment, including CD8+ T cells, FOXp3+ T cells (Tregs), and CD163+ M2 macrophages (TAMs) (Supplementary Fig. S4). The numbers of CD8+ T cells, FOXp3+ T cells, and CD163+ macrophages are shown in Fig. 4A. CD8+ T cells were the most prevalent tumor-infiltrating inflammatory/immune cells in our cohort. In the multivariate analysis, vascular invasion and FOXp3+ T cells were shown to be an independent prognostic factor for OS but not DFS (Supplementary Tables S3, S4). As expected, high CD8+ T cells were significantly associated with longer DFS (*p-*value = 0.016), whereas high CD163+ macrophages were significantly associated with worse OS (*p-*value = 0.041) (Supplementary Fig. S5). Surprisingly, FOXp3+ T cells were significantly associated with both better OS (*p-*value = 0.046) and DFS (*p-*value = 0.001) (Supplementary Fig. S5). Therefore, high CD8+/high FOXp3+/low CD163+ was associated with longer survival, which was interpreted as a favorable immune signature in our CCA cohort.

### Necroptosis activation is positively correlated with a favorable immune signature

We next investigated the relationship between necroptosis activation and different types of inflammatory/immune cells. As previously shown, it seems likely that MLKL and necroptosis activation (indicated by pMLKL) were differentially associated with survival rates of patients. We therefore also compared the correlation of necroptosis activation (pMLKL) and MLKL with tumor-infiltrating inflammatory/immune cells. Interestingly, pMLKL was positively correlated with CD8+ T cells (r = 0.281, *p-*value = 0.011) and FOXp3+ T cells (r = 0.253, *p-*value = 0.022) (Fig. 4B). It is also worth mentioning that we observed a relationship between CD8+ T cell infiltration in the tumor central area with pMLKL-positive staining, with increased pMLKL-positive staining being significantly associated with high CD8+ T cell abundance in the intratumoral infiltration (*p*-value = 0.006) (Fig. 4C, Supplementary Fig. S6). Conversely, MLKL was negatively correlated with CD8+ T cells (r = − 0.224, *p-*value = 0.041) and positively correlated with CD163+ macrophages (r = 0.263, *p-*value = 0.014) (Fig. 4B). These results demonstrated that necroptosis activation (pMLKL) was positively correlated with a favorable immune signature (high CD8+/high FOXp3+/low CD163+), whereas MLKL was positively associated with an unfavorable immune signature (low CD8+/low FOXp3+/high CD163+).

### A favorable immune/MLKL signature is correlated with longer disease-free survival

To be more precise and specific, a signature model based on expression profiles of MLKL and tumor-infiltrating inflammatory/immune cells were constructed. CCA patients with a high CD8+/high FOXp3+/low CD163+/low MLKL expression profile were classified as a favorable immune/MLKL signature. On the other hand, patients with a low CD8+/low FOXp3+/high CD163+/high MLKL expression profile were classified as an unfavorable immune/MLKL signature. Kaplan–Meier survival curves demonstrated that a favorable immune/MLKL signature was significantly associated with better DFS (*p-*value = 0.023, HR = 3.926) with higher hazard ratios when compared with a single prognostic factor (high CD8+/high FOXp3+/low CD163+, *p*-value = 0.008, HR = 3.347; MLKL, *p*-value = 0.016, HR = 2.117) (Fig. 4D). The median of DFS in patients with a favorable immune/MLKL signature was 15 months compared with that of 9 months for patients with an unfavorable immune/MLKL signature (Fig. 4D).

### Necroptosis activation is positively correlated with an immune checkpoint—PD-L1 expression

**Figure 5:**
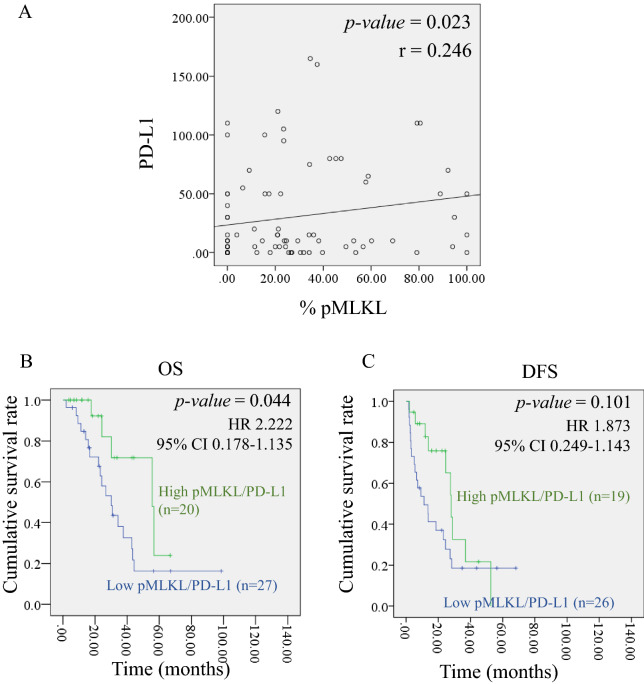
The relationship between necroptosis activation and immune checkpoint (PD-L1) expression. (**A**) The correlation analysis of pMLKL and PD-L1. Kaplan–Meier (**B**) overall survival (OS) curve and (**C**) disease-free survival (DFS) curve stratified by two groups of pMLKL and PD-L1 expression (high pMLKL/high PD-L1 and low pMLKL/low PD-L1).

A combination of immune checkpoint inhibitors with necroptosis treatment has been shown to enhance survival rates in an experimental mouse model^15,16^. PD-L1 expression on tumor cells—a well-known immune checkpoint—was analyzed by IHC staining (Supplementary Fig. S7A). PD-L1 was immunolocalized in both cytoplasm and plasma membrane in which in some patients, the membrane localization was predominantly observed (Supplementary Fig. S7A). The number of patients with an H-score above the median (10.00) who were interpreted as having a high PD-L1 expression was 49.9% (Supplementary Fig. S7B). There was no significant association between PD-L1 expression and survival rates for both OS and DFS in CCA patients (Supplementary Fig. S7C). Nevertheless, there was a trend of high PD-L1 associated with better OS and DFS. The median of OS in patients with high and low PD-L1 was 56 and 38 months, respectively (Supplementary Fig. S7C). Comparably, the median of DFS in patients with high and low PD-L1 was 28 and 14 months, respectively (Supplementary Fig. S7C). We further investigated the relationship of necroptosis activation (pMLKL) with PD-L1 expression in CCA patients. To our knowledge, this is the first study to reveal a positive correlation of pMLKL and PD-L1 expression in tumor cells (r = 0.246, *p-*value = 0.023) (Fig. 5A). Furthermore, we subdivided patients into two groups based on the combined status of pMLKL and PD-L1 expression. Interestingly, Kaplan–Meier survival curves showed that patients with high pMLKL/high PD-L1 had a longer OS (*p*-value = 0.044, HR = 2.22) (Fig. 5B,C). The median of OS in patients with high pMLKL/high PD-L1 and low pMLKL/low PD-L1 was 55 and 30 months, respectively (Fig. 5B). In line with these results, although without reaching statistical significance, the median of DFS in patients with high pMLKL/high PD-L1 and low pMLKL/low PD-L1 was 28 and 11 months, respectively (Fig. 5C). Altogether, our results demonstrated that necroptosis activation was positively correlated with PD-L1 expression, and patients with high pMLKL and high PD-L1 had a longer OS.

### Necroptosis activation in CCA cells promotes cytokine expression

**Figure 6:**
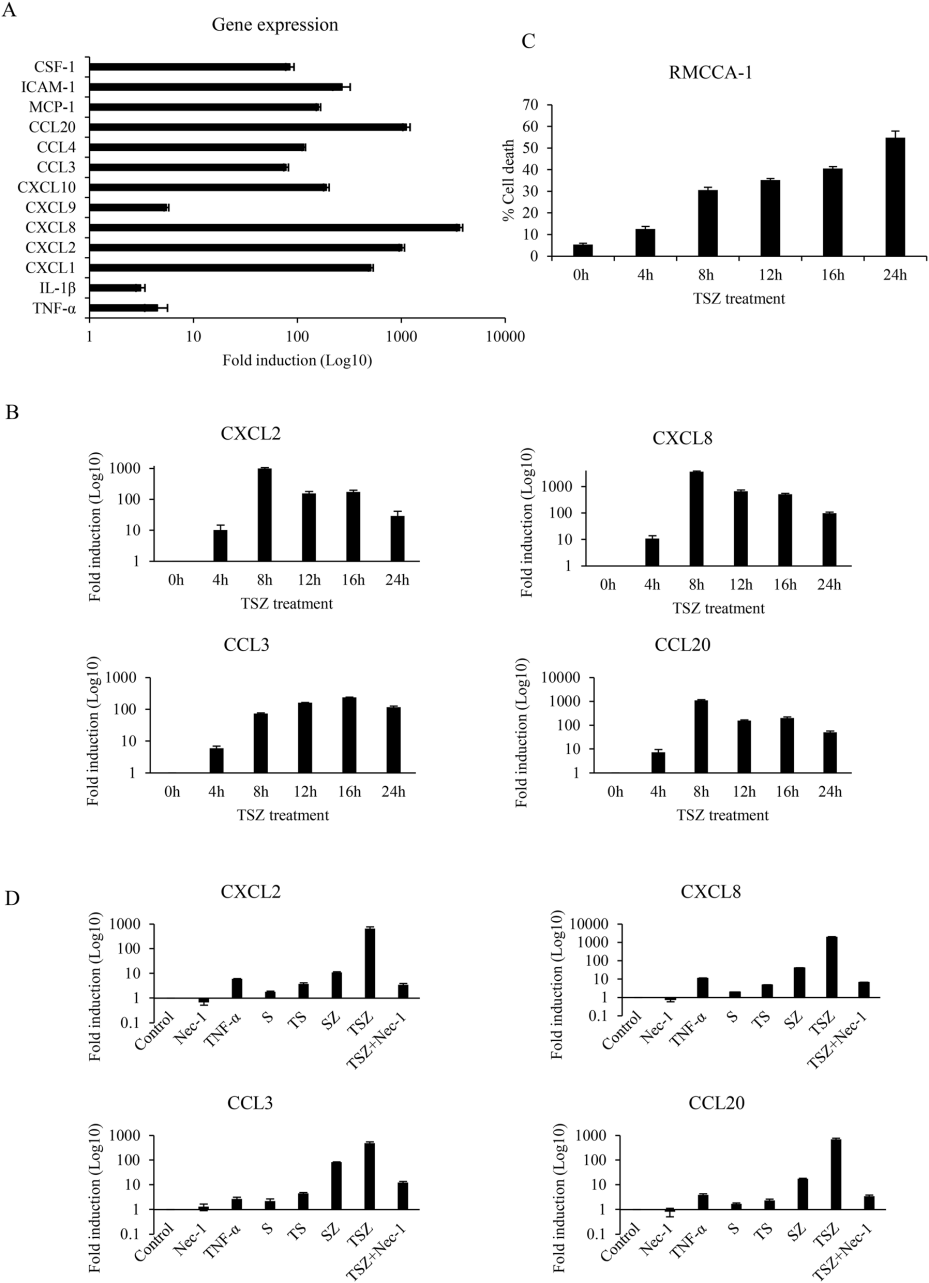
Tumor necroptosis increases proinflammatory cytokine and chemokine expression. (**A**) RMCCA-1 cells were treated with TSZ for 8 h. The transcriptional change of TNF-α, IL-1β, CXCL1, CXCL2, CXCL8, CXCL9, CXCL10, CCL3, CCL4, CCL20, MCP-1, ICAM-1, and CSF-1 were measured by qRT-PCR. (**B**) The time-course analysis of the selected chemokine gene expression, including CXCL2, CXCL8, CCL3, and CCL20 following TSZ treatment in RMCCA-1 (**C**) The percentage of cell death after stimulation with TSZ for indicated time points using AnnexinV/PI staining and analyzed by flow cytometry. **(D**) RMCCA-1 cells were treated with TNF-α or SM-164 (S) or SM-164 and zVAD-fmk (SZ) or apoptosis induction (TS). The cells were also pretreated with necrostatin-1 (Nec-1), a necroptosis inhibitor, followed by TSZ treatment for 24 h.

Our results indicated that necroptosis activation is correlated with a favorable immune signature, particularly intratumoral CD8+ T cell infiltration in CCA patients. Necroptosis has been shown to create an inflammatory tumor microenvironment and intratumoral infiltration of CD8+ T cells^28^, most likely mediated through cell-autonomous production of cytokines and releasing of DAMPs^13,29^. We then investigated whether necroptosis activation in CCA cells can promote proinflammatory cytokine and chemokine gene expression. We first analyzed the transcriptional changes of proinflammatory cytokines and chemokines, including TNF-α, IL-1β, CXCL1, CXCL2, CXCL8, CXCL9, CXCL10, CCL3, CCL4, CCL20, MCP-1, ICAM-1, and CSF-1 in necroptotic cells. RMCCA-1, a necroptosis sensitive human CCA cell line expressing RIPK1, RIPK3, and MLKL was stimulated with TNF-α (T), SM-164 (S), and a pan-caspase inhibitor zVAD-fmk (Z) (TSZ), a well-known necroptosis inducer, to induce necroptosis^25^. The mRNA expression of proinflammatory cytokines and chemokines was differentially upregulated in RMCCA-1 following TSZ treatment for 8 h (Fig. 6A). Interestingly, the most significant upregulated genes during necroptosis were CXCL1, CXCL2, CXCL8, CXCL10, CCL4, CCL20, MCP-1, and ICAM-1, all of which have been shown to be involved in immune cell infiltration and T cell trafficking. The time-course analysis of the selected chemokine genes’ expressions, including CXCL2, CXCL8, CCL3, and CCL20 in RMCCA-1 stimulated with TSZ, showed that most of the mRNA expression of the gene set was significantly upregulated after TSZ treatment for 8 h and gradually declined (Fig. 6B). The increased expression of the mRNA level was correlated with the phosphorylation of MLKL as shown previously^25^ and occurred when the cells began to die (Fig. 6C). In contrast to necroptosis (TSZ) treatment, the expression of these chemokines was much weaker in RMCCA-1 treated with TNF-α or SM-164/zVAD-fmk (SZ) alone, or not induced with S alone or with apoptosis induction (TNFα/SM-164, TS) (Fig. 6D). Moreover, a necroptosis inhibitor, necrostatin-1 (Nec-1), dramatically inhibited the expression of these chemokines during necroptosis induction, as seen in Fig. 6D. Taken together, these results suggested that proinflammatory cytokines and chemokines were selectively induced upon necroptosis activation in CCA cells.

### Necroptotic-conditioned medium stimulates Jurkat T cells to induce PD-L1 expression in CCA cells

**Figure 7:**
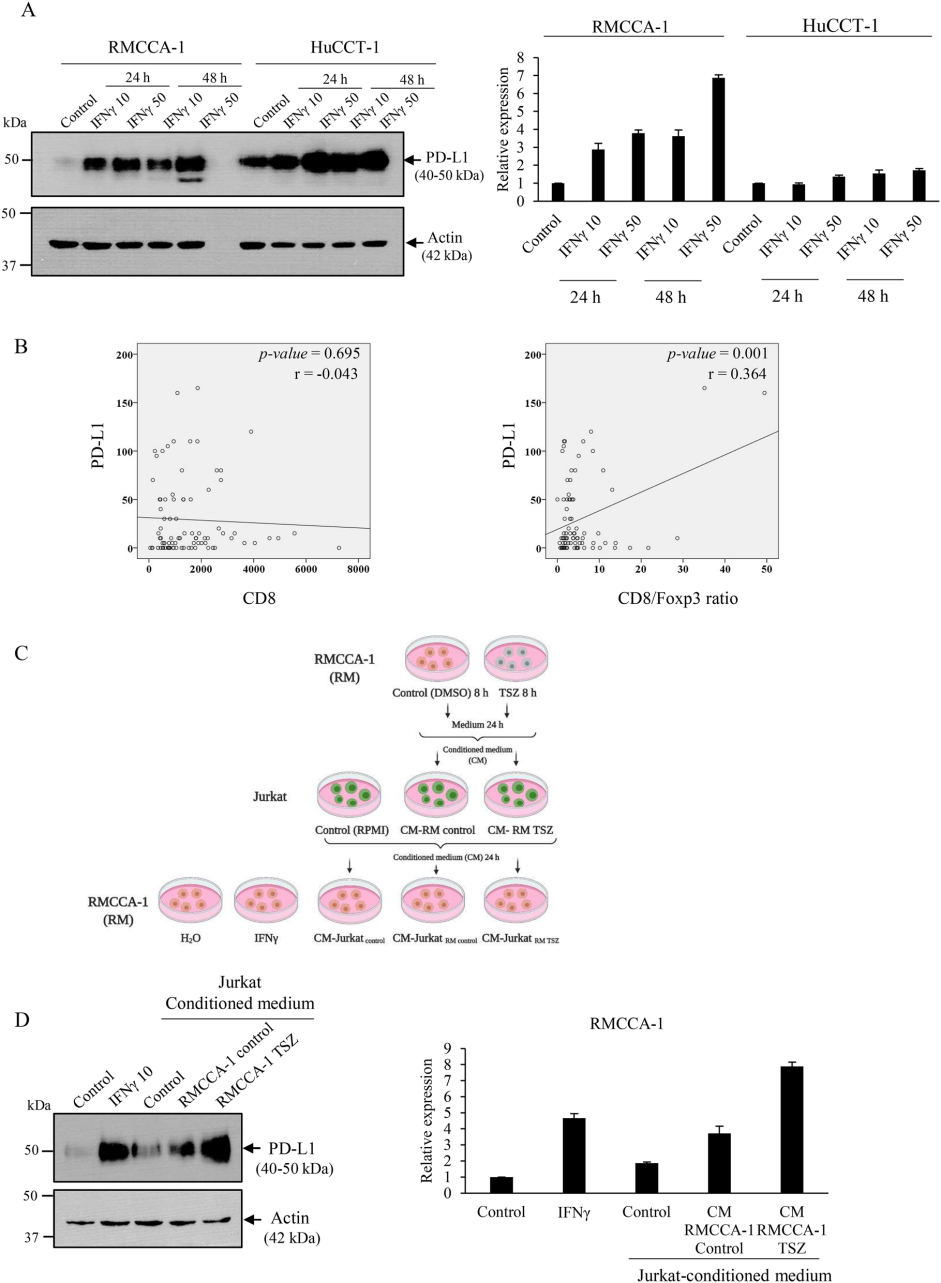
Tumor necroptosis promotes T cell activation to upregulate PD-L1 expression in CCA cells. (**A**) Western blot analysis for PD-L1 expression of RMCCA-1 and HuCCT-1 treated with IFNγ at 10 and 50 ng/ml for indicated time points. (**B**) The correlation analysis of CD8+ T cells or CD8+ T cells/Foxp3+ ratio with PD-L1 expression in CCA cells (**C**) Schematic of treatment conditions, RMCCA-1 cells were treated with DMSO or TSZ for 8 h and conditioned medium (CM) were collected. Jurkat cells were stimulated with the conditioned medium and the conditioned medium from each treatment conditions were collected as shown. (**D**) Western blot analysis for PD-L1 expression of RMCCA-1 treated with conditioned media from Jurkat.

Our findings have revealed that necroptosis activation in tumor cells was positively correlated with tumor PD-L1 expression in CCA patients. Therefore, we attempted to uncover the association of necroptosis activation and PD-L1 expression in an in vitro cell model. Because T cell-derived interferon gamma (IFNγ) is well known to upregulate PD-L1 expression in various cancer cells^30^, to determine the effect of IFNγ on PD-L1 expression in CCA cell lines, we treated RMCCA-1 and HuCCT-1 with 10 and 50 ng/ml of IFNγ for 24 and 48 h, and PD-L1 expression was examined by Western blot analysis. The results demonstrated that PD-L1 expression in both RMCCA-1 and HuCCT-1 was dramatically increased in response to IFNγ treatment (Fig. 7A). The expression of PD-L1 upon stimulation with IFNγ was higher in RMCCA-1 than HuCCT-1 (Fig. 7A). In addition, IHC analysis in CCA patients demonstrated that although intratumoral CD8+ T cell infiltration did not correlate with PD-L1 expression in CCA cells, the higher ratio of CD8+ T cells to FOXp3+ T cells (suppressor T cells) was positively correlated with higher PD-L1 expression in tumor cells (r = 0.364, *p*-value = 0.001) (Fig. 7B). These results indicate a relationship between intratumoral CD8+ T cell infiltration and PD-L1 expression in CCA patients^31^. All together, we therefore hypothesized that the release of DAMPs from tumor necroptosis in the CCA tumor microenvironment might promote CD8+ T cell activation, which in turn induces PD-L1 expression in CCA cells. To this end, we collected the conditioned medium of necroptotic RMCCA-1 cells (TSZ treatment), and subsequently, the conditioned medium was incubated with Jurkat^32^—a human T cell line—for an additional 24 h. Naïve RMCCA-1 cells were then incubated with the conditioned medium collected from Jurkat cells stimulated with the necroptotic-conditioned medium for 24 h to investigate the PD-L1 expression level. The experimental setup is shown in Fig. 7C. Interestingly, PD-L1 expression was markedly increased in RMCCA-1 cells stimulated with the conditioned medium from Jurkat cells that had been stimulated with the necroptotic-conditioned medium (Fig. 7D). RMCCA-1 treated with IFNγ was used as a positive control (Fig. 7D). Altogether, these results suggest that tumor necroptosis increases PD-L1 expression in CCA cells, most likely mediated through tumor necroptosis-induced T cell activation.

## Discussion

Necroptosis-based cancer therapy has been proposed as a potential novel strategy that could enhance cancer immunotherapy, but its functional roles in tumorigenesis have remained unknown because both pro- and anti-tumoral effects have been reported as described above. Our studies provided a list of novel findings on necroptosis in human malignancy and demonstrated the differential roles between MLKL and necroptosis activation (pMLKL) in CCA patients. MLKL, which might function independently of its role in necroptosis, was an unfavorable prognostic factor for patients with CCA. Both pMLKL and RIPK1–RIPK3 interaction were detected in human CCA primary tissues. Of particular interest, pMLKL status was significantly positively correlated with a clinically favorable immune signature (high CD8+/high FOXp3+/low CD163+), whereas MLKL was positively associated with a clinically unfavorable immune signature (low CD8+/low FOXp3+/high CD163+). In addition, this is the first study to demonstrate the relationship between necroptosis activation and PD-L1 expression and revealed that their associations also impact patient survival rates. In vitro experiments demonstrated that tumor necroptosis promoted the expression of proinflammatory cytokines and chemokine genes and induced Jurkat T cells to upregulate PD-L1 expression in CCA cells. Collectively, the results of the present study suggested that in contrast to MLKL, necroptosis activation may have anti-tumorigenic roles, and necroptosis-based treatment in combination with immune checkpoint inhibitors, could provide more efficient treatment for CCA patients.

In our previous study analyzed from the TCGA database, we demonstrated the upregulation of key necroptotic proteins, including RIPK1, RIPK3, and MLKL in CCA primary tissues compared to normal bile ducts^25^. We confirmed the expression of RIPK3 and MLKL in CCA clinical specimens by IHC staining. Our study was the first to demonstrate that MLKL expression was significantly upregulated in CCA clinical specimens. In agreement with our study, high expression of MLKL was also reported in PDA^20^. Interestingly, patients with high MLKL were associated with worse survival rates; this result was not consistent with other malignancies, including PDA^33^, gastric cancer^34^, colon cancer^35^, ovarian cancer^36^, cervical cancer^37^, and breast cancer^38^ in which MLKL expression level was decreased in tumor tissues, and low MLKL was associated with worse survival rates in those cancers. Hence, those studies suggested that MLKL might play an anti-tumorigenic role, probably through necroptosis activation. Further studies are required to explore the functional roles of MLKL in CCA, either through necroptosis activation or independent of its role in necroptosis.

Necroptosis has been implicated in various malignancies. However, growing evidence in most of the studies relied on experimental knockout of key necroptotic proteins, including RIPK3 and MLKL or chemical inhibitors in experimental animal models^39^. In addition, the clinical relevance of necroptosis activation in human clinical samples has remained largely unknown. To the best of our knowledge, only few studies have reported necroptosis activation by pMLKL detection in human malignancies^19,40,41^. We used the same antibody obtained from Abcam (ab187091) for IHC^19,40^ and IF^41^ staining of pMLKL as in those studies above, but in our present study, pMLKL immunoreactivity was clearly located in the cell membrane in both HT-29 cell block, a positive control, and human CCA primary tissues compared to an elevated cytoplasmic staining or no clear staining pattern reported in the other studies aforementioned. In addition, no research has been reported regarding the application of an in situ PLA assay to determine RIPK1 and RIPK3 interaction in human clinical specimens. In the present study, RIPK1–RIPK3 interaction and pMLKL were also detected in the very same tumor cells, indicating the activation of necroptosis in CCA. It is true that the functional roles of necroptosis in cancer have remained not necessarily established and, therefore, we further explored the potential impact of necroptosis activation on the survival of CCA patients in this study. In contrast to MLKL expression, we revealed that patients with high pMLKL tended to survive for longer. However, one of the limitations in our present study was a rather unsatisfactory number of cases available for examination, thereby resulting in an inadequate statistical power. These results were inconsistent with a study in colon and esophageal patients, in which a relatively high level of pMLKL was reported to be associated with adverse clinical outcome of the patients^40^. The results of a recent study in a breast cancer mouse model were also consistent with those above, in which pMLKL was increased during the progression of mouse breast cancer, indicating that necroptosis might be involved in cancer progression and metastasis^19^. Therefore, further investigations are required to clarify the roles of necroptosis in CCA patients.

Necroptosis associated with marked inflammatory response has been reasonably postulated to modulate the tumor microenvironment. However, none has demonstrated an association between the activation of intratumoral necroptosis and inflammatory/immune cell infiltration in human malignancies. Our study was consistent with a previous study in CCA patients, in which their preliminary data demonstrated a direct association between RIPK3 expression and the intratumoral infiltration of CD8+ T cells (only abstract available^42,43^). In contrast, the analysis of PDA in an experimental mouse model with RIPK3 deletion demonstrated a significantly increased number of CD4+ and CD8+ T cells but decreased number of FOXp3+, TAMs, and myeloid-derived suppressor cells (MDSC), indicating that deletion of RIPK3 was indeed associated with increased T cell infiltration and reduced immunosuppressive myeloid cells^20^. Therefore, this study is the first one to demonstrate the correlation of necroptosis activation and tumor-infiltrating immune cells in the actual tumor microenvironment of human malignancies.

MLKL was a clinically unfavorable independent prognostic marker and inversely correlated with a clinically favorable immune cell signature (high CD8+/high FOXp3+/low CD163+). Conversely, necroptosis activation (pMLKL) was positively correlated with a clinically favorable immune cell signature in our study of CCA patients. These results demonstrated the differential roles of MLKL and its role in necroptosis activation in CCA patients. Accumulating evidence indicates the newly identified functions of MLKL beyond necroptosis, including regulating the generation of intraluminal and extracellular vesicles^44,45^ and endothelial cell adhesion gene expression^46^; however, the functional roles and a deeper mechanistic understanding of non-necroptotic functions of MLKL in cancers, including CCA, require further investigation^47^. Consistent with other studies in these patients, infiltration of CD8+ T cells and CD163+ M2 macrophages were both significantly associated with longer and shorter patient survival in our CCA cohorts, respectively^4,5,48–50^ but the FOXp3+ regulatory T cells reported to suppress immune responses against tumor cells and to be correlated with poor clinical outcome has remained an opposing finding compared to other types of cancers^51^. Previous studies in two different cohorts of extrahepatic CCA patients also reported a discrepancy regarding the potential roles of FOXp3+ T cells; the results of our present study were consistent with those of Gosper et al.^4^ and also two recent reports in colorectal cancer patients^52,53^. FOXp3+ T cells can be further divided into subsets based on their expression levels of FOXp3+ and CD45RA indicating naïve and effector Treg T cells and non-suppressive T cells^54^. Therefore, the quantification of the percentage of non-suppressive T cells is likely to help clarify the clinical relevance of FOXp3+ in CCA and reduce the limitation in this study, although further investigations are necessary for clarification.

Necroptosis has recently emerged as a potential target of cancer therapy in conjunction with cancer immunotherapy. Necroptotic cancer cells can provide both tumor-specific antigens and DAMPs to dendritic cells, potentially also amplifying the process of antigen cross-priming and the activation of CD8+ cytotoxic T cells^13,55^. In this study, we firstly demonstrated a significant positive correlation between necroptosis activation (pMLKL) and infiltration of CD8+ T cells in human cancer patients. Interestingly, CD8+ T cell infiltration was observed in tumor areas with pMLKL-positive staining. Infiltration of CD8+ T cells, a key player in anti-tumor immunity, was significantly associated with a longer DFS, suggesting that necroptosis-based cancer therapy could be a potential therapeutic option for CCA patients, improving the clinical outcomes through activation of the immune system. We conducted in vitro experiments to further explore the association of necroptosis and intratumoral CD8+ T cell infiltration. Tumor necroptosis has been shown to create an inflammatory tumor microenvironment and intratumoral infiltration of CD8+ T cells^28^, probably mediated through cell-autonomous production of cytokines and release of DAMPs^13,29^. In agreement with a previous study^29^, we showed that necroptosis of CCA cells promoted the upregulation of proinflammatory cytokine and chemokine gene expression. The proinflammatory cytokines and chemokines, including CXCL1, CXCL2, CXCL8, CXCL10, CCL4, CCL20, MCP-1, and ICAM-1 have been shown to be involved in the recruitment of various immune cells including CD8+ T cells^56^. Therefore, the production of these cytokines and chemokines upon necroptosis activation in CCA cells might contribute to the recruitment of immune cells into the CCA tumor microenvironment, supporting the positive correlation of necroptosis activation and the infiltration of CD8+ T cells in CCA patients. Further studies in an immunocompetent animal model of CCA are required to clarify the association of tumor necroptosis and intratumoral CD8+ T cell infiltration in an in vivo setting.

**Figure 8:**
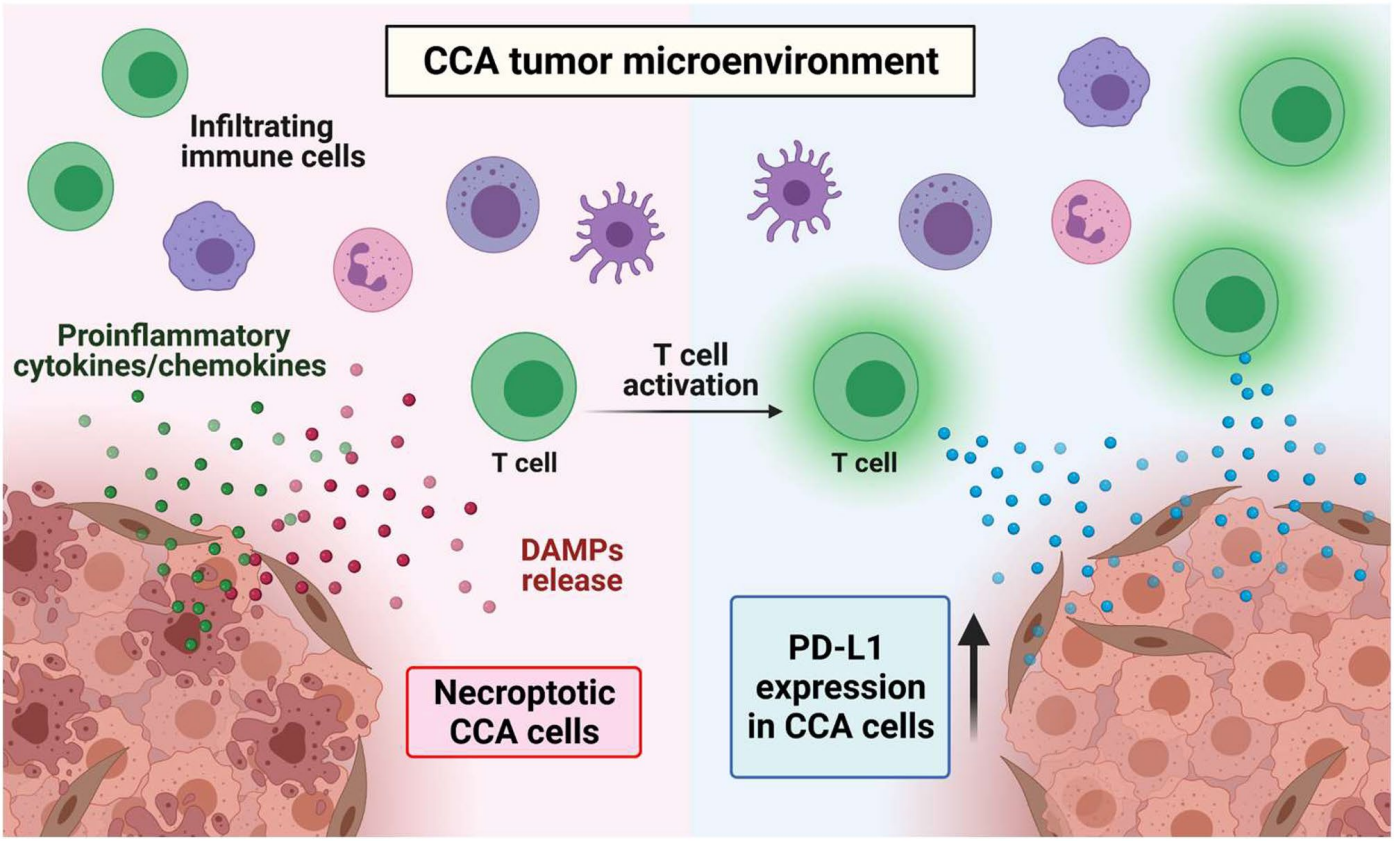
Proposed mechanisms of necroptosis activation-promoted immune cell infiltration and PD-L1 expression in cholangiocarcinoma. Necroptosis activation in CCA cells increases proinflammatory cytokine and chemokine production which creates inflammatory tumor microenvironment and recruits tumor-infiltrating immune cells and T cell trafficking to the tumor microenvironment. Moreover, the release of damage-associated molecular patterns (DAMPs) from the necroptotic CCA cells activate T cells, which in turn promote PD-L1 expression in CCA cells. *Created with BioRender.com

In addition, PD-L1 expressed on tumor cell membrane was also established a key factor, which could enable tumor cells to escape immune surveillance^57^. Therefore, targeting PD-L1 and its receptor—PD-1—using immune-modulating monoclonal antibodies has been increasingly administered in various human malignancies at clinical settings^58^. We firstly demonstrated that a subset of CCA patients was immunohistochemically positive for PD-L1. PD-L1 expression has been reported in CCA patients by different groups, but the results obtained demonstrated a contradictory association with survival of the patients, partly due to the different selection of PD-L1 antibody clones and evaluation criteria^50,59–65^. In this study, we analyzed PD-L1 expression using an E1L3N antibody clone, and PD-L1 expression was evaluated by the H-score method. Analysis of PD-L1 expression by the H-score in extrahepatic CCA was shown to reflect prognosis better than other evaluation methods^65^. In addition, a recent report regarding metastatic melanoma demonstrated that exosomal PD-L1 is associated with an anti-PD-1 response^66^, and another study revealed that the exosomal PD-L1 in the plasma of patients with non-small cell lung cancer was correlated with PD-L1 expression in tumor cells analyzed by the H-score^67^. Nivolumab treatment was recently reported to yield a prolonged response to immunotherapy in PD-L1-positive CCA patients^68^. We also firstly demonstrated in this study that tumor PD-L1 was positively correlated with necroptosis activation—pMLKL. Therefore, we attempted to further discover the possible underlying mechanisms of necroptosis activation and PD-L1 expression in in vitro settings. The upregulation of PD-L1 expression was reported to be dependent on IFNγ signaling in various cancer cell types^30^. Our studies in CCA cell lines were consistent with previous research in other cancers; we showed that PD-L1 expression was upregulated following IFNγ treatment in two CCA cell lines. These results suggest that CCA cells could respond to IFNγ-induced PD-L1 expression. CD8+ T cells represent one of the majority of cells that secrete IFNγ in the tumor microenvironment, a process that could promote PD-L1 expression in tumor cells^31^. We therefore hypothesized that the release of DAMPs from necroptotic tumor cells could activate T cells to promote PD-L1 expression in CCA cells. Interestingly, this is the very first study to reveal the interplay between the necroptosis of tumor cells and the upregulation of PD-L1 in tumor cells that is mediated through tumor necroptosis-stimulated T cell activation. However, the deeper underlying mechanisms of how tumor necroptosis-stimulated T cell activation promotes PD-L1 expression in CCA cells should be further investigated. Taken together, we therefore proposed that necroptosis in CCA cells through the activation of proinflammatory cytokine and chemokine gene expression could create an inflammatory microenvironment and recruit immune cells, including T cells, into the tumor microenvironment, and the release of DAMPs from the necroptotic tumor cells could activate T cells, which in turn promote PD-L1 expression in CCA cells (Fig. 8). In addition, a subgroup of patients who have high pMLKL and high PD-L1 had a longer OS. A combination of immune checkpoint inhibitors with necroptosis-based cancer treatment has been reported to promote durable tumor clearance when compared to a single treatment in experimental animal models^15,16^. Our findings regarding the association of high pMLKL and high PD-L1 and a clinically favorable OS in CCA patients therefore provided the first clinical relevance in human cancer patients and supported a recent study proposing the use of checkpoint blockade immunotherapy in cancer and CCA patients with an inflamed subtype^7,69^. Collectively, our findings raise the possibility of developing a necroptosis-based therapy in combination with immune checkpoint inhibitors for more efficient CCA therapy.

In conclusion, this is the first study in human cancer patients to systematically analyze and compare the associations of MLKL and necroptosis activation with tumor-infiltrating immune cells in the tissue CCA microenvironment and their impact on clinical outcomes of the patients themselves. Our findings demonstrated the differential associations of MLKL and necroptosis activation with a clinically favorable immune signature and survival and should contribute to a better understanding of the clinical significance of necroptosis activation and more likely a non-necroptotic function of MLKL. Together with the significant positive correlation between necroptosis activation and an immune checkpoint, the presence of high PD-L1 and high pMLKL in tumor cells were implicated in the longer OS. Moreover, our in vitro experiments further support the association of tumor necroptosis and intratumoral infiltration of CD8+ T cells and demonstrate the potential role of the relationship between tumor necroptosis and PD-L1 expression that is probably mediated through tumor necroptosis-promoted T cell activation. The results of our study, therefore, highlighted a novel therapeutic possibility for the combination of a necroptosis-based therapeutic approach with immune checkpoint inhibitors toward prolonging the survival time of CCA patients.

## Materials and methods

### Antibodies

Anti-RIPK3 (ab72106), anti-MLKL (ab184718), anti-pMLKL (ab187091), and anti-FOXp3 (ab20034) were purchased from Abcam (Cambridge, UK); anti-PD-L1 (E1L3N) was obtained from Cell Signaling Technology (Danvers, Massachusetts, USA); anti-RIPK1 (610459) was bought from BD Biosciences (San Jose, California, USA); anti-CD8 (M7103) was purchased from Dako (Agilent) (Santa Clara, California, USA); and anti-CD163 (NCL-L-CD163) was purchased from Leica Biosystems (Wetzlar, Germany).

### Patient selection and clinical data collection

Formalin-fixed and paraffin-embedded (FFPE) tissue specimens were retrieved from a total of 88 CCA patients (Intrahepatic CCA = 21 samples and Hilar CCA = 67 samples) who had undergone curative surgery at Tohoku University Hospital, Sendai, Japan between 2005 and 2015. Informed consent was obtained from all patients before surgery. Clinicopathological parameters of the individual patients examined are summarized in Supplementary Table S1. None of the patients received neoadjuvant therapy before surgery. The study protocol was approved by IRB of Tohoku University School of Medicine, Sendai, Japan. All experiments were performed in accordance with relevant guidelines and regulations.

### Preparation of cell block HT-29 cells

The cell blocks were prepared for optimization and validation of pMLKL staining and in situ PLA assay. HT-29 cells were solidified using iPGell (Genostaff, Tokyo, Japan) according to the manufacturer’s instructions. Briefly, cells were collected and fixed with 10% neutral-buffered formalin for 10 min. Solutions A and B were added to solidify the cells. The clot was gently removed from the tube and transferred to a tissue cassette. Subsequently, a tissue cassette was soaked into ethanol and xylene and finally embedded in paraffin.

### Immunohistochemical (IHC) staining

Tissue sections (3 µm) were cut, and tissues were deparaffinized and hydrated in xylene and ethanol, respectively, then autoclaved for 5 min in an antigen retrieval solution—sodium citrate buffer (pH 6.0). The slides were blocked with 3% hydrogen peroxide at room temperature for 10 min and then incubated with primary antibodies at 4 °C overnight. Subsequently, the slides were incubated with biotin-streptavidin horseradish peroxidase-conjugated secondary antibody (Nichirei Bioscience, Japan) at room temperature for 1 h; after that, the antigen–antibody complexes were visualized with 3,3′-diaminobenzidine tetrahydro-chloride solution and then counterstained with hematoxylin. Tissue sections of normal human liver or pancreas were used as a positive control for RIPK3 staining, whereas normal tonsil was the positive control for MLKL staining. For the negative controls, the primary antibodies were omitted in the procedure of immunostaining.

### Scoring of immunoreactivity

Histopathological and immunohistochemical analysis were determined by 5 of the authors (TL, PA, MH, KM, and HU) using multi-headed light microscopy (BX50; Olympus, Tokyo, Japan). All tissue sections were scored in a semiquantitative manner. For semiquantitative analysis of immunoreactivity of RIPK3, MLKL, and PD-L1, the modified H-score was employed. The H-score was defined by a > 500 tumor cell count from 3 different representative fields, giving a possible range of 0–300. The H-score was calculated from the formula (%Strong × 3) + (%Moderate × 2) + %Weak. H-scores of 0–50, 51–100, 101–200, and 201–300 indicated that RIPK3 and MLKL expression intensities were negative, low, moderate, and strong, respectively. Low and high RIPK3, MLKL, and PD-L1 expression were divided based on the median H-score of all specimens.

### Evaluation of pMLKL staining and quantitative evaluation of immune cell infiltration

The pMLKL was classified by labeling index using the following formula: Labeling index (%) = number of pMLKL-positive cells × 100/number of total cells counted. Inflammatory/immune cell infiltration was initially determined by H&E staining. To quantify the tumor-infiltrating inflammatory/immune cells, three non-overlapping fields with high numbers of tumor-infiltrating immune cells (hot spots) were selected (Supplementary Fig. S4). The stained slides were scanned and digitally converted into virtual slides using NanoZoomer (Hamamatsu Photonics K.K., Japan). Inflammatory/immune cells were counted under high power magnification (× 400) by two of the investigators (TL and HU) using digital image analysis (Halo imaging analysis software; Indica Labs, Corrales, New Mexico, USA). For statistical analyses, low and high CD8 + T cells, FOXp3 T cells, and CD163 + macrophages were grouped using the median as a cutoff.

### Immunofluorescence (IF) staining

Paraffin sections (3 μm) were dewaxed with xylene and ethanol. Antigen retrieval was performed by heating the slides in an autoclave at 121 °C for 5 min in citrate buffer (pH 6.0). The slides were incubated for 30 min at room temperature with blocking solution and were then incubated with primary antibodies at 4 °C overnight. The slides were subsequently incubated with Alexa Fluor 488 anti-rabbit and Alexa Fluor 555 anti-mouse secondary antibodies (Invitrogen, Carlsbad, California, USA) at room temperature for 1 h. The reacted slides were then mounted with mounting medium with DAPI (Invitrogen, Carlsbad, California, USA).

### In situ proximity ligation assay (in situ PLA)

Duolink in situ PLA kit (Olink Bioscience, Uppsala, Sweden) was used to detect RIPK1–RIPK3 interaction. Paraffin sections (3 μm) were dewaxed and rehydrated; antigen retrieval was then carried out in Tris–EDTA buffer pH 9.0 at 125 °C for 20 min in an autoclave. The slides were incubated at 37 °C for 30 min with blocking solution and were then incubated with primary antibodies (mouse anti-RIPK1 and rabbit anti-RIPK3) at 4 °C overnight. The slides were then incubated with PLA PLUS and MINUS probes for mouse and rabbit at 37 °C for 1 h. Ligation-Ligase solution was added and incubated at 37 °C for 30 min. Amplification polymerase solution was subsequently added and incubated at 37 °C for 100 min. The slides were mounted with mounting medium and stained with DAPI.

### Cell lines and culture

HuCCT-1, a human CCA cell line, was obtained from the Japanese Collection of Research Bioresources (JCRB) Cell Bank, Osaka, Japan. RMCCA-1, a human CCA cell line, was developed from Thai patients with CCA^70^. Both HuCCT-1 and RMCCA-1 were cultured in HAM’s F-12 medium (HyClone Laboratories, Logan, Utah, USA) supplemented with 10% fetal bovine serum (Sigma, St Louis, Missouri, USA) and 1% Penicillin–Streptomycin (HyClone Laboratories, Logan, Utah, USA). Jurkat, a human T cell line, was obtained from the American Type Culture Collection (ATCC, Manassas, VA, USA). Jurkat was cultured in RPMI (HyClone Laboratories, Logan, Utah, USA) supplemented with 10% fetal bovine serum (Sigma, St Louis, Missouri, USA) and 1% Penicillin–Streptomycin (HyClone Laboratories, Logan, Utah, USA). All cell lines were cultured in a humidified incubator at 37 °C with 5% CO_2_. All cell lines were tested for mycoplasma contamination and were mycoplasma free.

### Necroptosis treatment and collection of conditioned medium

Necroptosis induction in RMCCA-1, RMCCA-1 was pretreated with Smac mimetic, SM-164 (10 nM) and zVAD-fmk (20 μM) for 30 min. Then, the cells were treated with TNF-α (10 ng/ml). Necrostatin-1 (Nec-1), a necroptosis inhibitor, was used at a concentration of 30 μM. For the collection of conditioned medium from RMCCA-1, necroptosis was induced by TSZ treatment for 8 h. After that, the culture medium was removed and replaced with fresh serum-free RPMI. Cells were incubated for an additional 24 h. The conditioned medium was collected and centrifuged at 3000 rpm for 5 min to remove cell debris prior to using it to stimulate Jurkat T cells. Resting Jurkat T cells (1 × 10^6^ cells) were incubated with 1 ml of the conditioned medium of TSZ- (necroptosis) or DMSO-treated (control) RMCCA-1 for 24 h. The conditioned medium was collected as described above. Naïve RMCCA-1 (2 × 10^5^ cells) were incubated with 1 ml of the conditioned medium obtained from Jurkat T cells stimulated with the necroptotic (TSZ) or control (DMSO) conditioned media or RPMI medium. The experimental setup is shown in Fig. 7C.

### RNA preparation, reverse transcription, and real-time PCR

Total RNA was extracted from cells using TRIzol reagent (Invitrogen, Carlsbad, California, USA). RNA (1 μg) was reverse-transcribed using RevertAid Reverse Transcriptase (Thermo Fisher Scientific, Waltham, Massachusetts, USA) with oligo (dT) 18 primer according to the manufacturer's protocol. Real-time PCR was performed by using an iTaq™ universal SYBR® Green supermix (Bio-Rad, Hercules, California, USA) following the manufacturer’s instructions. All PCR primers used in this study are listed in Supplementary Table S5. PCR cycling parameters were 95 °C for 30 s, followed by 40 cycles of 95 °C for 15 s and 57 °C for 30 s, and fluorescence signals were measured in real time. GAPDH was used as an internal control to normalize the amount of total RNA added to each reaction, and relative gene expressions are presented with the ΔΔCt method. The results are expressed as fold induction over control cells.

### Western blot analysis

Cells were lysed in RIPA buffer (Merck Millipore, Darmstadt, Germany) with a proteinase inhibitor cocktail (Roche, Mannheim, Germany). Total proteins were separated by 10% gel SDS-PAGE and subsequently transferred onto PVDF membranes. The membranes were incubated with PD-L1 antibody (E1L3N) (Cell Signaling Technology, Danvers, Massachusetts, USA). Actin (4970) (Cell Signaling Technology, Danvers, Massachusetts, USA) was used as a loading control. The proteins were visualized by enhanced chemiluminescence according to the manufacturer’s instructions (Bio-Rad, Hercules, California, USA). All Western blots shown are representative of at least three independent experiments.

### Statistical analysis

All statistical analyses were conducted using the software package SPSS for Windows. Independent prognostic factors were identified by multivariate analysis with a Cox-regression model. DFS and OS of patients were defined as the interval time from the date of resection to the date of cancer recurrence and the date of death from cancer, respectively. The cumulative survival times of patients were estimated by the Kaplan–Meier method, and statistical differences between two groups were calculated by a log-rank test. Pearson’s correlation methods were performed to determine whether there was a positive or negative correlation between two groups. The Mann–Whitney *U*-test was used for two-group comparisons. All *p*-values less than 0.05 were considered statistically significant.

## Supplementary Information

Supplementary Information.

## Data Availability

All data generated or analyzed during this study are included in this published article and the Supplementary Information files.
