# Supplementary material for: Tumor necroptosis is correlated with a favorable immune cell signature and programmed death-ligand 1 expression in cholangiocarcinoma
Source: Sci Rep. 2021 Jun 3;11:11743. doi: 10.1038/s41598-021-89977-9 (PMC8175514; doi:10.1038/s41598-021-89977-9)
Supplement: Supplementary file 1 — Supplementary Information. [file 41598_2021_89977_MOESM1_ESM.pdf]

Supplementary Information

Tumor necroptosis is correlated with a favorable immune cell signature and programmed death-ligand 1 expression in cholangiocarcinoma

Thanpisit Lomphithak[1], Perawatt Akara-amornthum[1], Keigo Murakami[2], Masatoshi Hashimoto[2], Hajime Usubuchi[2], Erina Iwabuchi[2], Michiaki Unno[3], Zhenyu Cai[4], Hironobu Sasano[2], Siriporn Jitkaew[5]*

***Corresponding author**:

Dr. Siriporn Jitkaew

Address: Age-Related Inflammation and Degeneration Research Unit, Department of Clinical Chemistry, Faculty of Allied Health Sciences, Chulalongkorn University, Bangkok, 10330, Thailand.

Email address: Siriporn.ji@chula.ac.th

Tel: (66) 022181081, Fax: (66) 022181082

Table of Content:

1. Supplementary Tables 1-5
2. Supplementary Figures 1-8

Supplementary Table S1. Clinical backgrounds of patients enrolled in this study.

**Gender**	N (%)
Male	52 (59%)
Female	36 (41%)
**Age (years)**	
Median	67 (38-85)
**Grading**	
Well differentiated	13 (14.8%)
Moderately differentiated	73 (83%)
Poorly differentiated	2 (2.2%)
**TNM Stage**	
I	2 (2%)
II	27 (31%)
III	21 (24%)
IV	34 (39%)
Missing	4 (4%)
**Tumor size (mm)**	
>= 35	39 (44.3%)
< 35	49 (55.7%)
**Perineural Invasion**	
Present	65 (73.9%)
None	23 (26.1%)
**Vascular Invasion**	
Present	66 (75%)
None	22 (25%)
**Lymph node invasion**	
Present	62 (70.5%)
None	26 (29.5%)
**Neoadjuvant therapy**	
None	88 (100%)

Supplementary Table S2. **Univariate and Multivariate Cox proportional analysis for OS: Key necroptotic proteins and clinicopathological features.**

Factor	Univariate				Multivariate			
	95% CI		HR	*p*-value	95% CI		HR	*p*-value
**Gender**	0.401	1.381	0.744	0.349				
**Age (MED = 67 years)**	0.606	2.041	1.112	0.731				
**Type**	0.909	3.331	1.740	0.095				
**HistoGradeEU**	0.488	2.309	1.061	0.880				
**Tumor size (MED = 35 mm)**	0.771	2.670	1.435	0.254				
**RIPK3 ($MED_{H\text{-}Score}$ = 160)**	0.442	1.495	0.813	0.506				
**MLKL ($MED_{H\text{-}Score}$ = 120)**	1.040	4.883	2.254	0.039	0.972	4.601	2.114	0.059
**Vascular Invasion**	0.273	0.991	0.520	0.047	0.975	3.576	1.867	0.060
**Neural/Perineural invasion**	0.417	1.606	0.819	0.561				
**Lymph node invasion**	0.410	1.529	0.791	0.486				
**TNM stage**	0.805	4.348	1.870	0.146				

Supplementary Table S3. Univariate and Multivariate Cox proportional analysis for DFS: Inflammatory/immune cells infiltration and clinicopathological features.

Factor	Univariate				Multivariate			
	95% CI		HR	*p*-value	95% CI		HR	*p*-value
**Gender**	0.745	2.177	1.274	0.376				
**Age (MED = 67 years)**	0.797	2.602	1.440	0.227				
**Type**	1.322	4.173	2.349	0.004	0.052	3.583	2.311	0.437
**Vascular Invasion**	0.273	0.848	0.481	0.011	0.027	1.817	4.490	0.161
**Neural/Perineural invasion**	0.354	1.102	0.625	0.104				
**Lymph node invasion**	0.364	1.110	0.636	0.111				
**TNM stage**	0.547	1.625	0.942	0.831				
**CD8 (MED= 1133 cells)**	0.304	0.893	1.920	0.018	0.719	2.479	1.335	0.360
**CD163 (MED= 216 cells)**	0.690	1.970	1.166	0.566				
**FOXp3 (MED= 378 cells)**	0.229	0.690	2.518	0.001	0.889	3.362	1.728	0.107

Supplementary Table S4. Univariate and Multivariate Cox proportional analysis for OS: Inflammatory/immune cells infiltration and clinicopathological features.

Factor	Univariate				Multivariate			
	95% CI		HR	*p*-value	95% CI		HR	*p*-value
**Gender**	0.401	1.381	0.744	0.349				
**Age (MED = 67 years)**	0.606	2.041	1.112	0.731				
**Type**	0.909	3.331	1.740	0.095				
**Vascular Invasion**	0.273	0.991	0.520	0.047	1.048	3.926	2.028	0.036
**Neural/Perineural invasion**	0.417	1.606	0.819	0.561				
**Lymph node invasion**	0.410	1.529	0.791	0.486				
**TNM stage**	0.805	4.348	1.870	0.146				
**CD8 (MED= 1133 cells)**	0.441	1.527	0.651	0.534				
**CD163 (MED= 216 cells)**	1.015	3.595	0.821	0.045	1.119	4.178	2.162	0.022
**FOXp3 (MED= 378 cells)**	0.286	0.998	1.910	0.049	0.918	3.307	1.743	0.085

Supplementary Table S5. List of primer sequences used for RT-qPCR

Gene	Forward primer	Reverse primer
TNF-α	GCCCATGTTGTAGCAAACCC	CTGATGGTGTGGGTGAGGAG
IL-1β	TGAGCTCGCCAGTGAAATGA	CATGGCCACAACAACTGACG
CXCL1	AAGCTTGCCTCAATCCTGCA	CCTCTGCAGCTGTGTCTCTC
CXCL2	GAAAGCTTGTCTCAACCCCG	CCTCTGCAGCTGTGTCTCTC
CXCL8	CCAGGAAGAAACCACCGGAA	TGGGGTGGAAAGGTTTGGAG
CXCL9	GCTGGTTCTGATTGGAGTGC	TTTTCTCGCAGGAAGGGCTT
CXCL10	GAACCTCCAGTCTCAGCACC	GCAGGTACAGCGTACAGTTCT
CCL3	ACATTCCGTCACCTGCTCAG	CTGGCTGCTCGTCTCAAAGT
CCL4	TGAAGCTCTGCGTGACTGTC	GCTTCCTCGCGGTGTAAGAA
CCL20	GTGCTGCTACTCCACCTCTG	GCATTGATGTCACAGCCTTCA
MCP-1	TCAAACTGAAGCTCGCACTCT	GGTGACTGGGGCATTGATTG
ICAM-1	TGATGGGCAGTCAACAGCTA	GCAGCGTAGGGTAAGGTTCT
CSF-1	ACACCATGCGCTTCAGAGAT	CATAGAAAGTTCGGACGCAGG
GAPDH	ACATCGCTCAGACACCATGG	ACCAGAGTTAAAAGCAGCCCT

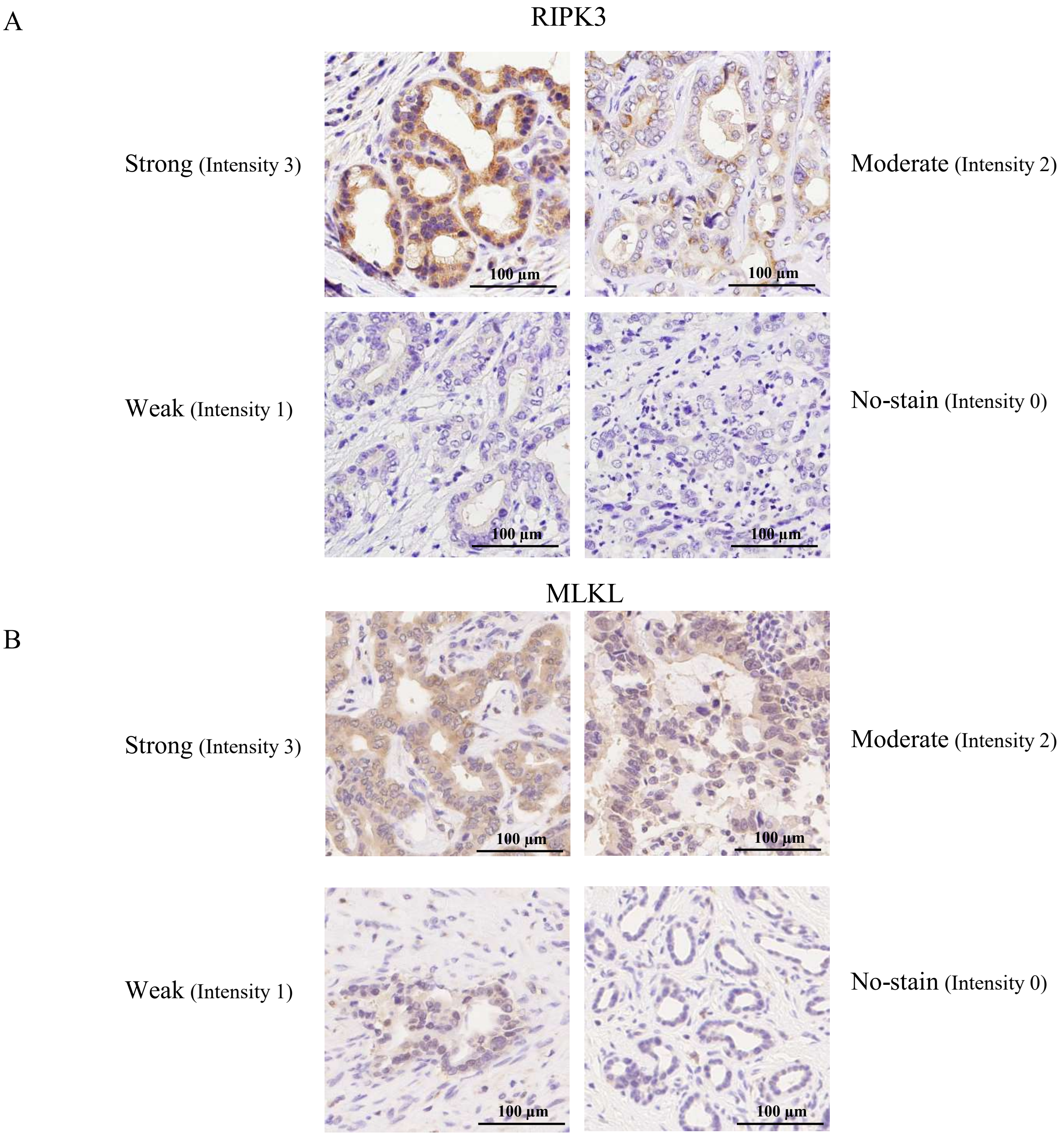

C

**Median of H-score**		**n**	**Area**		***p*-value**
			**Tumor area**	**Tumor adjacent normal area**	
RIPK3	All CCA	88	158.48	154.86	0.767
	Hilar CCA	67	165.44	144.69	0.024
	Intrahepatic CCA	21	123.37	181.05	0.011
	Cholangiocytes	8	167.50		
	Liver cells	8	185.00		
MLKL	All CCA	88	122.52	28.77	1.3001E-31
	Hilar CCA	67	114.78	22.04	1.1394E-24
	Intrahepatic CCA	21	150.91	47.89	7.8992E-10
	Cholangiocytes	4	30.00		
	Liver cells	4	0.00		

Suppl. Fig. 1 RIPK3 and MLKL expression in primary CCA tissues. The representative immunohistochemical staining of (A) RIPK3 and (B) MLKL scored as 3 for strong staining, 2 for moderate staining, 1 for weak staining and 0 for no staining. (B) The median of H-score staining in CCA primary tissues (tumor tissues and adjacent).

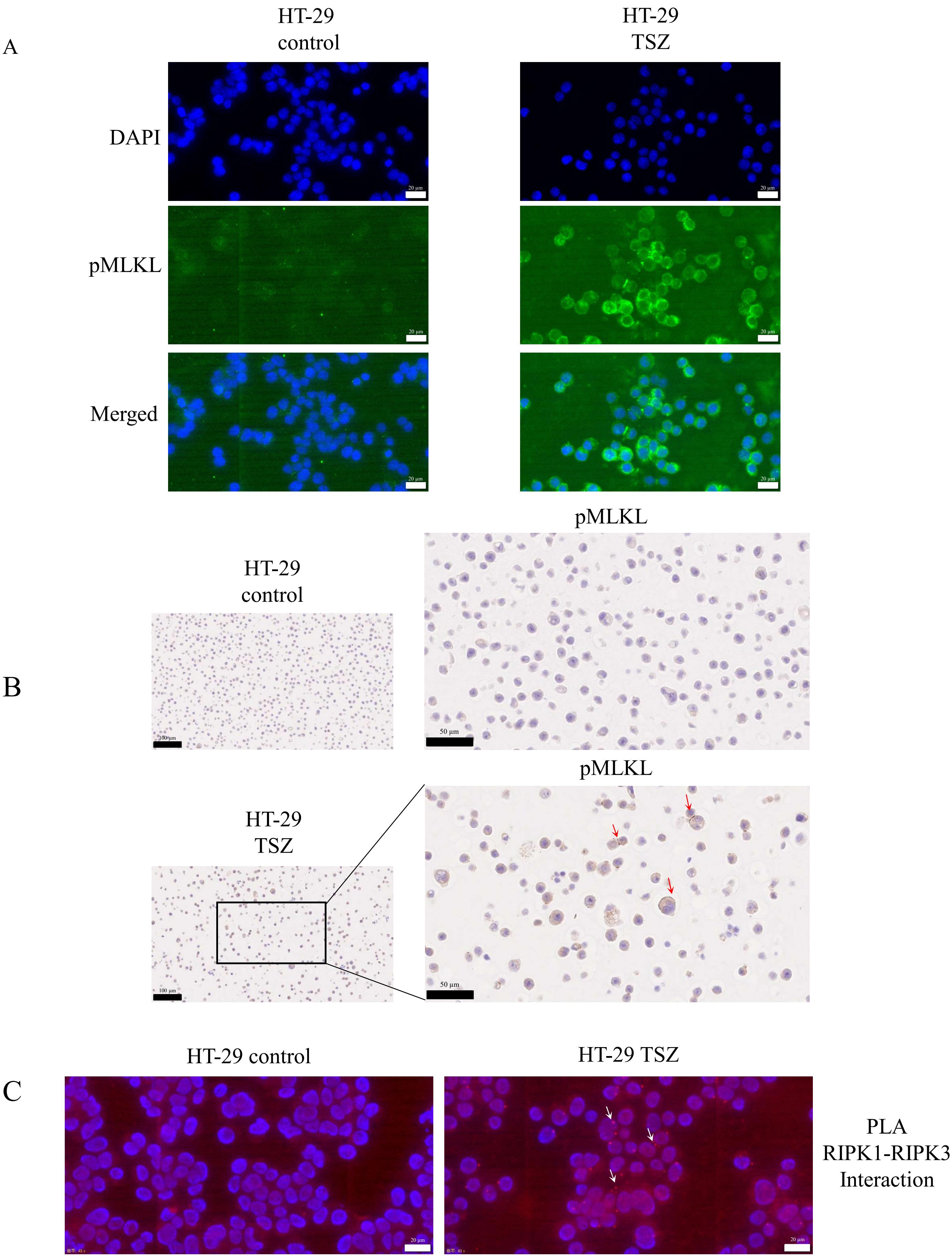

Suppl. Fig. 2 The optimization of pMLKL antibody and PLA assay for necroptosis detection. The representative (A) immunofluorescence staining, (B) immunohistochemical staining of pMLKL and (C) RIPK1-RIPK3 interaction by PLA assay in HT-29 cells treated with necroptotic stimuli (TNF-α/Smac mimetic/zVAD-fmk; TSZ) compared with control cells.

A

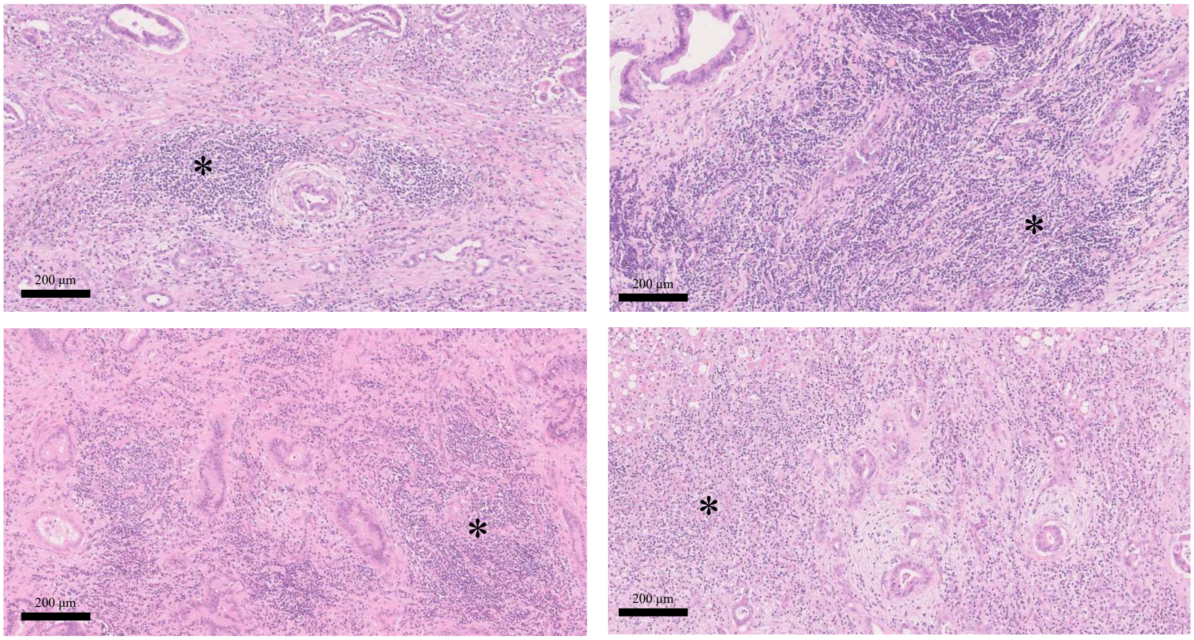

B

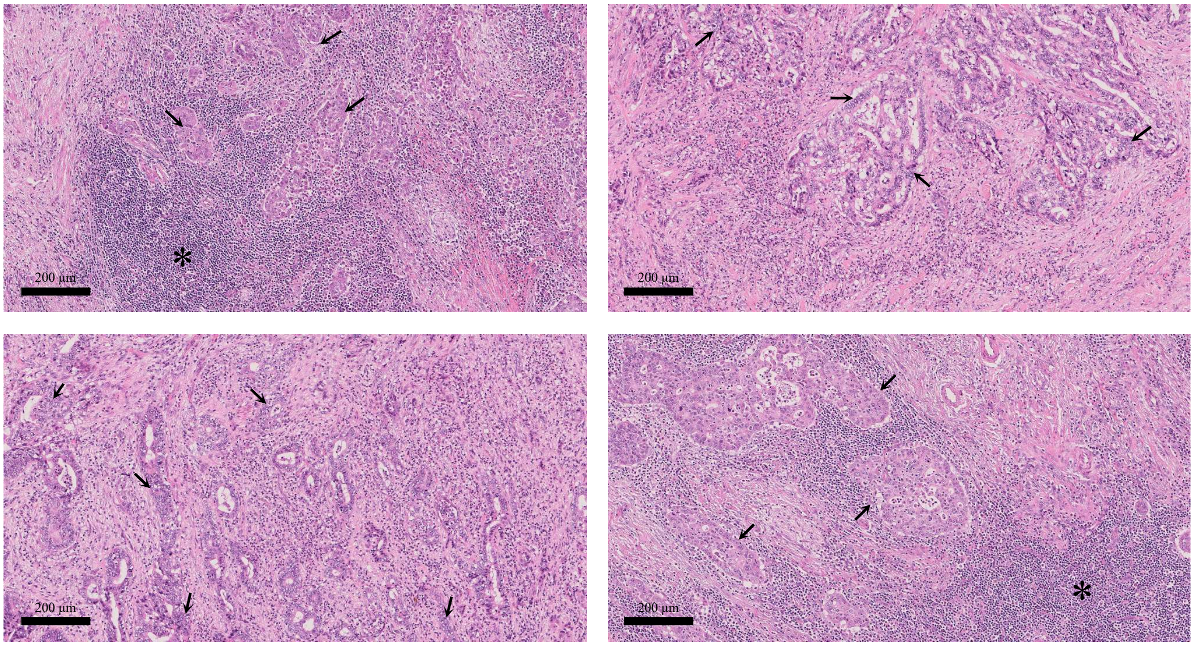

Suppl. Fig. 3 The infiltration of inflammatory/immune cells in CCA tumor microenvironment. The representative hematoxylin and eosin (H&E) staining of CCA primary tissues indicating a high abundance infiltration of inflammatory/immune cells in tumor central area (A) and tumor necrotic area (B) in CCA tumor microenvironment. Asterisks (*) indicate the high abundance of inflammatory/immune cells infiltrated around tumor. Arrows indicate tumor necrosis.

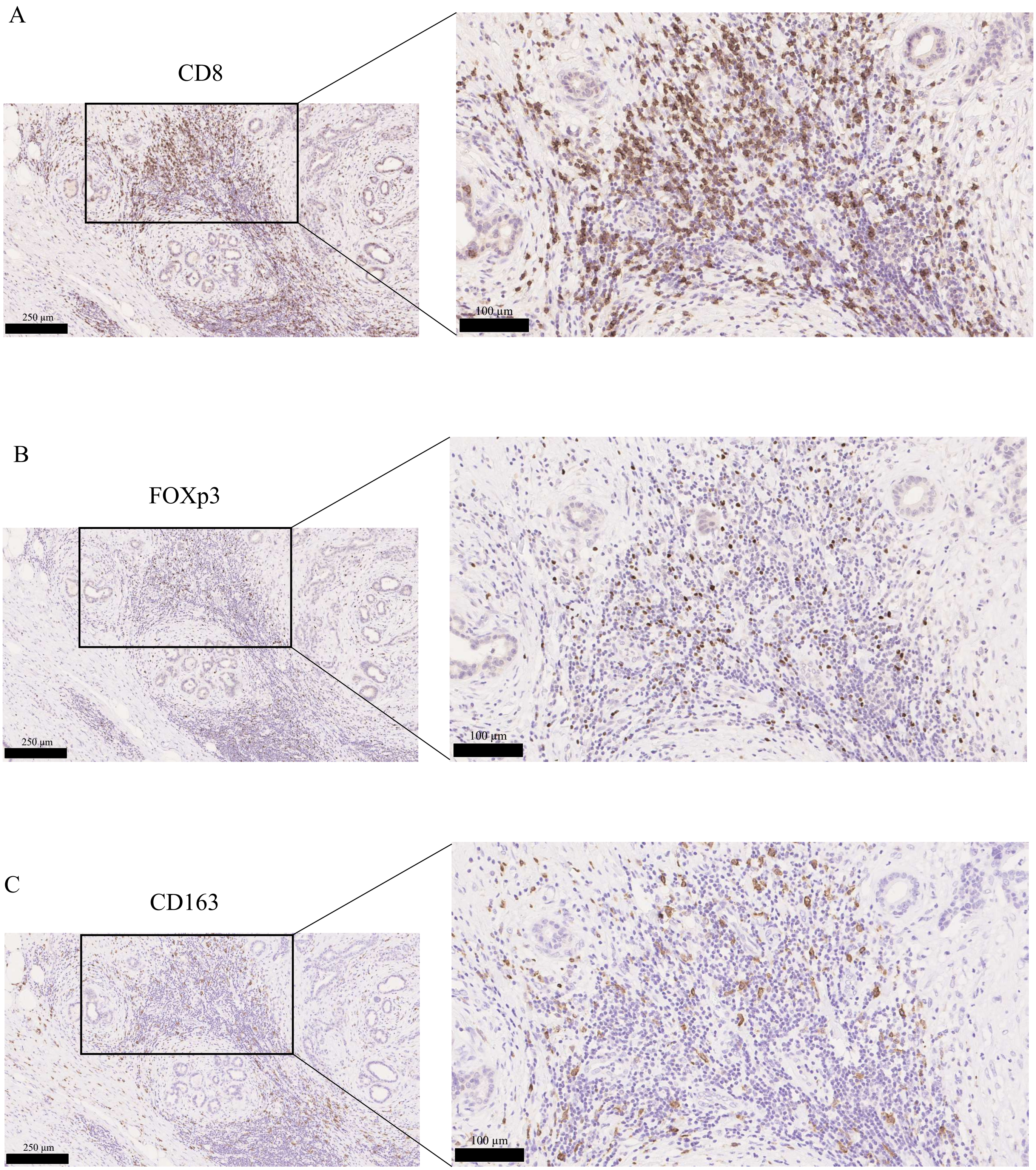

Suppl. Fig. 4 The representative immunohistochemical staining of (A) CD8+ T cells, (B) FOXp3+ T cells, and (C) CD163+ M2 macrophages.

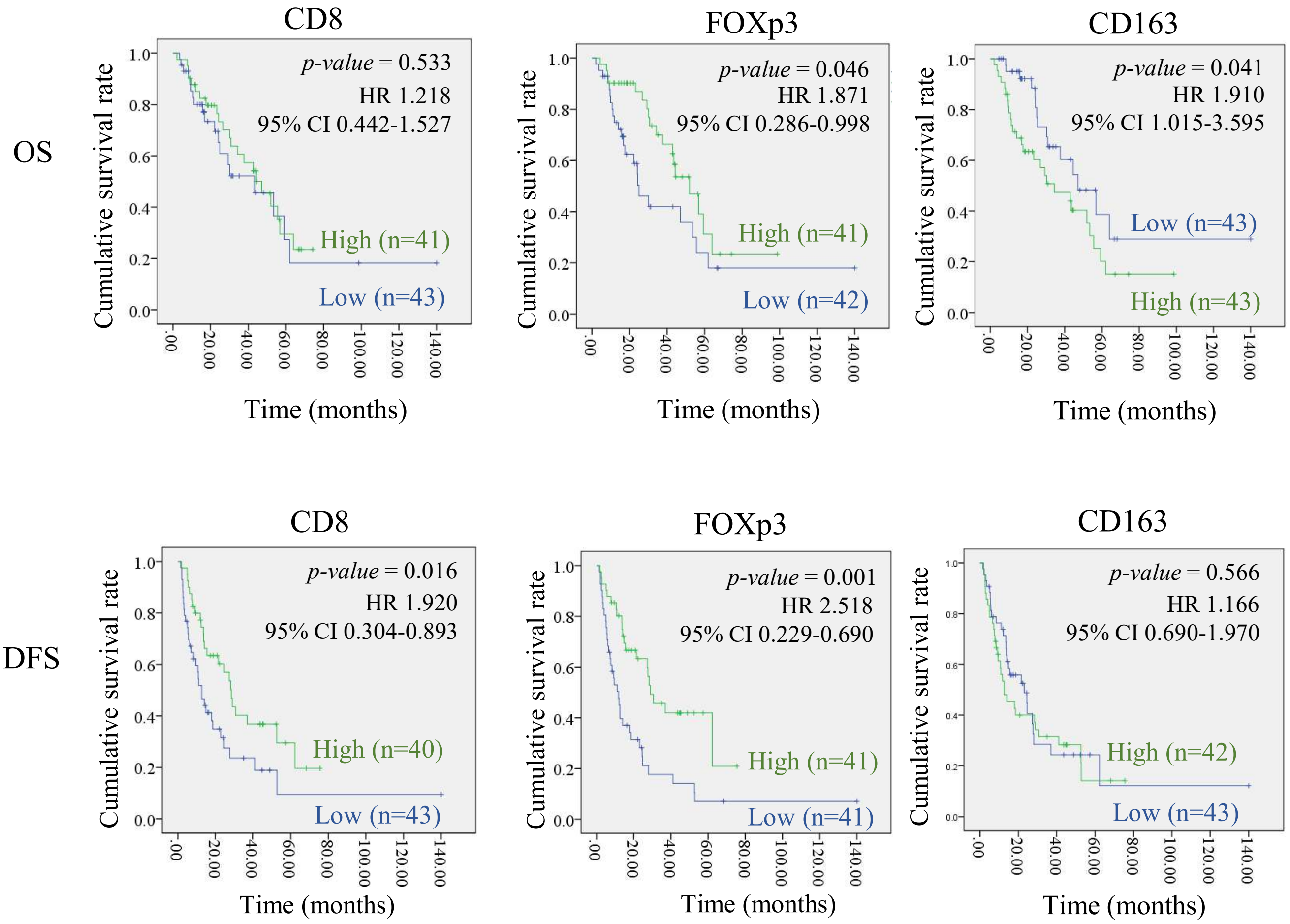

Suppl. Fig. 5 Kaplan-Meier overall survival (OS) and disease-free survival (DFS) curves stratified by two groups of CD8+ T cells, FOXp3+ T cells, and CD163+ M2 macrophages infiltration (high and low)

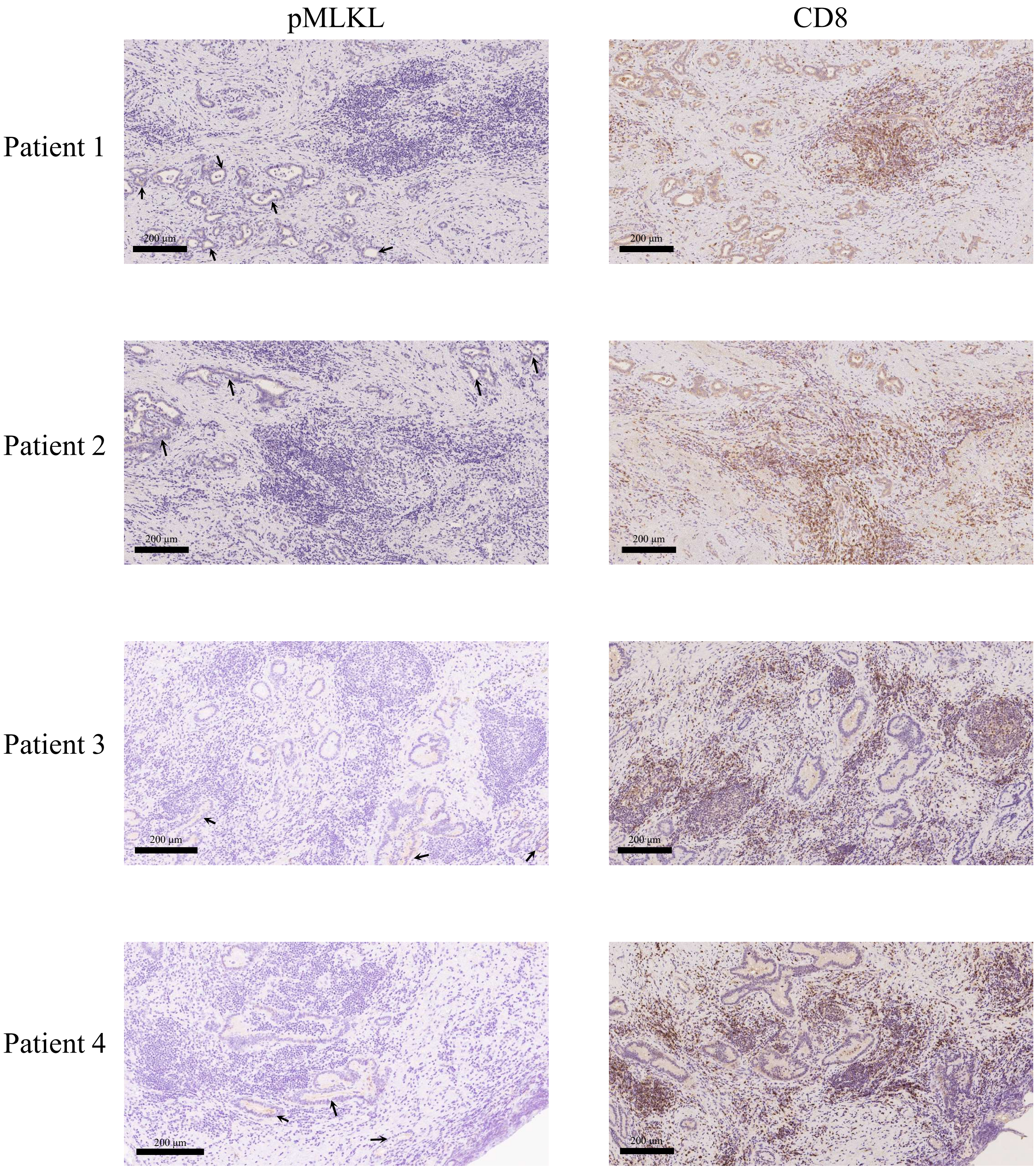

Suppl. Fig 6. The intratumoral infiltration of CD8+ T cells into pMLKL positive CCA cells. The representative immunohistochemical staining images showing the intratumoral infiltration of CD8+ T cells into pMLKL positive CCA cells. Arrows indicate pMLKL positive CCA cells.

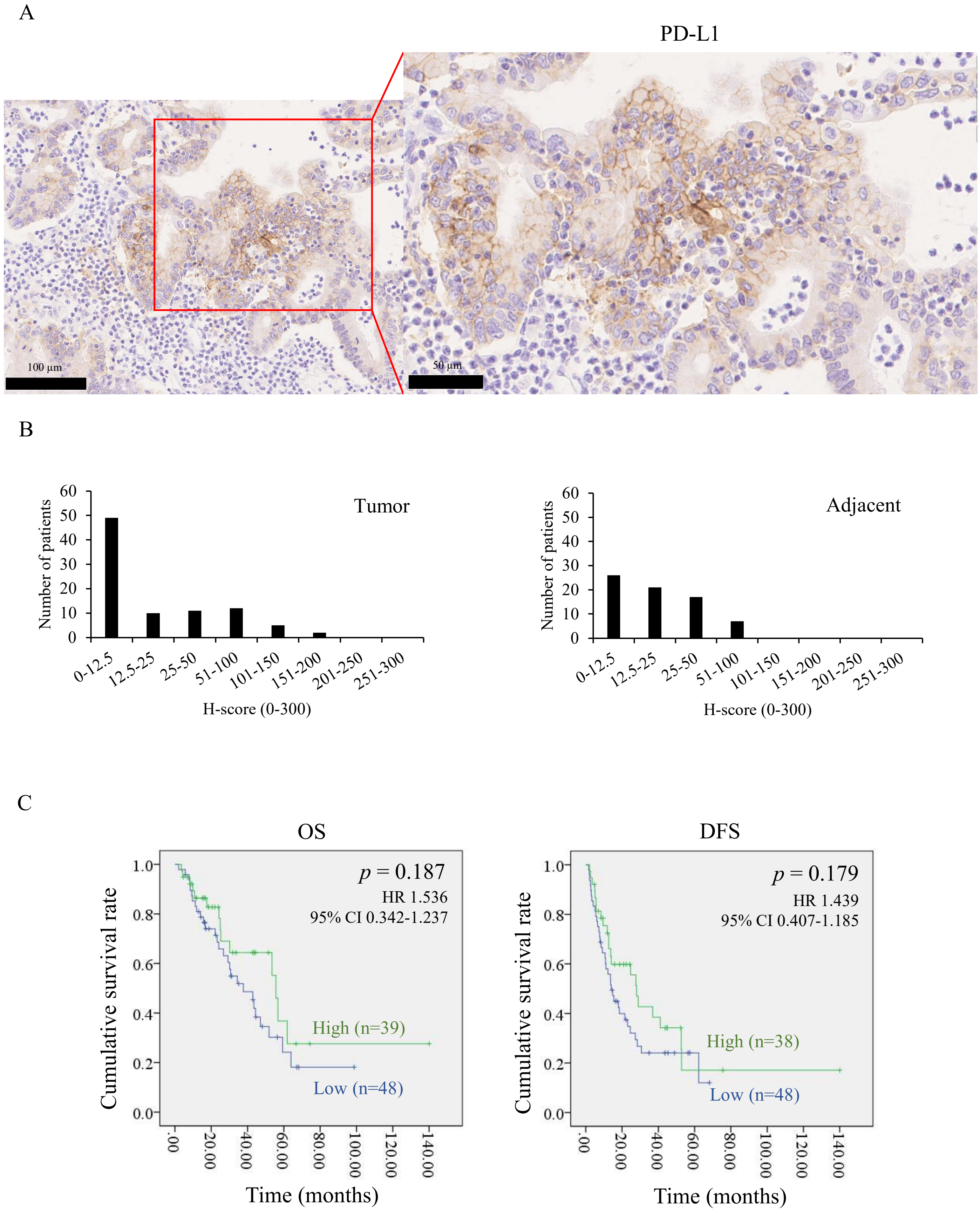

Suppl. Fig. 7 PD-L1 expression in primary CCA tissues. (A) The representative immunohistochemical staining of PD-L1 in primary CCA tissues. (B) Distributions of PD-L1 expression according to H-score of tumor area and adjacent. (C) Kaplan-Meier survival analysis of PD-L1 for overall survival (OS) and disease-free survival (DFS), respectively.

Fully unedited images for Figure 7A

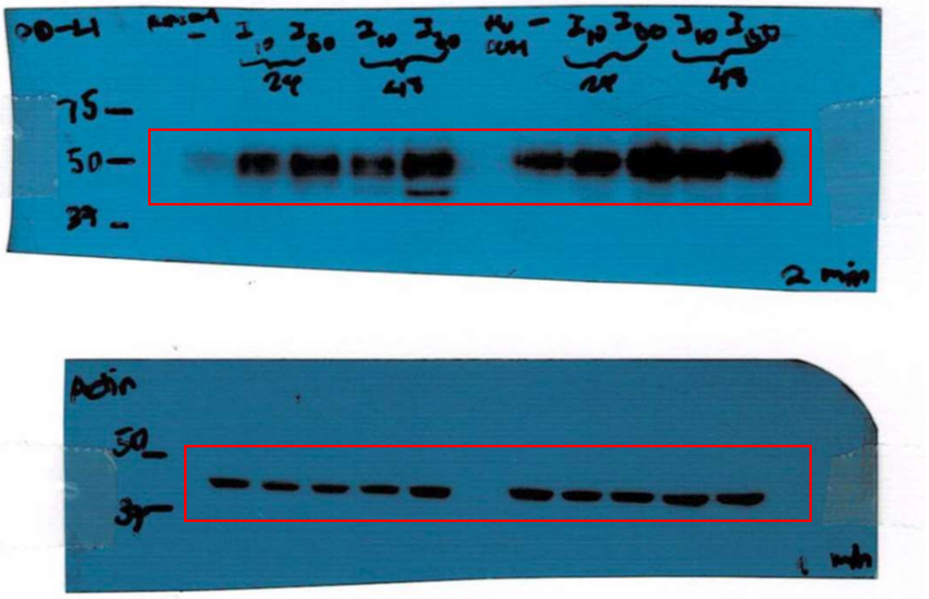

Fully unedited images for Figure 7D

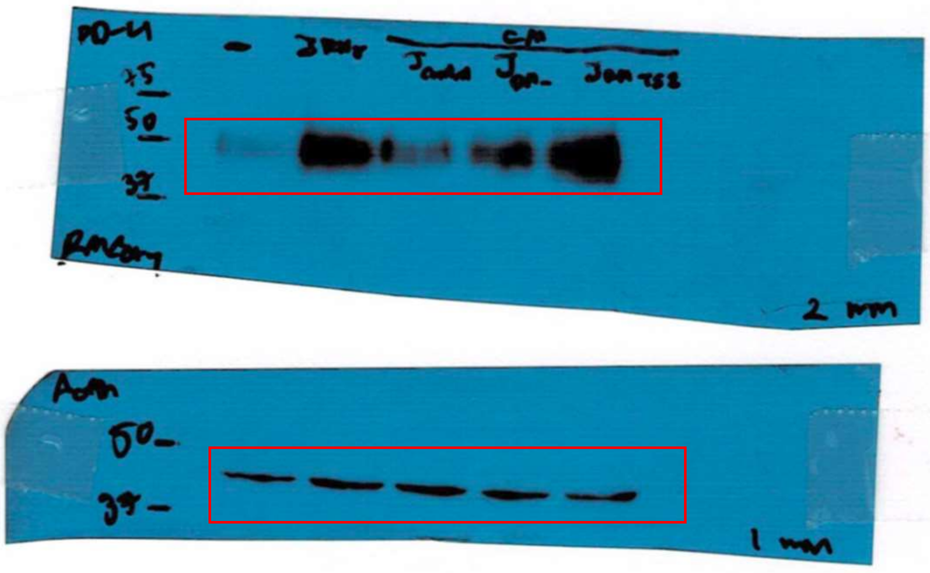

Suppl. Fig. 8 Fully unedited images for Figure 7A and 7B
